# Decoding the role of gut microbiota in Alzheimer’s pathogenesis and envisioning future therapeutic avenues

**DOI:** 10.3389/fnins.2023.1242254

**Published:** 2023-09-18

**Authors:** Junyi Liang, Bin Liu, Xiaohong Dong, Yueyang Wang, Wenhui Cai, Ning Zhang, Hong Zhang

**Affiliations:** ^1^Heilongjiang University of Traditional Chinese Medicine, Harbin, Heilongjiang, China; ^2^Heilongjiang Jiamusi Central Hospital, Jiamusi, Heilongjiang, China

**Keywords:** Alzheimer’s disease, gut microbiota, gut-brain axis mechanism, memory, inflammation, traditional Chinese medicine, treatment research advancements

## Abstract

Alzheimer’s disease (AD) emerges as a perturbing neurodegenerative malady, with a profound comprehension of its underlying pathogenic mechanisms continuing to evade our intellectual grasp. Within the intricate tapestry of human health and affliction, the enteric microbial consortium, ensconced within the milieu of the human gastrointestinal tract, assumes a role of cardinal significance. Recent epochs have borne witness to investigations that posit marked divergences in the composition of the gut microbiota between individuals grappling with AD and those favored by robust health. The composite vicissitudes in the configuration of the enteric microbial assembly are posited to choreograph a participatory role in the inception and progression of AD, facilitated by the intricate conduit acknowledged as the gut-brain axis. Notwithstanding, the precise nature of this interlaced relationship remains enshrouded within the recesses of obscurity, poised for an exhaustive revelation. This review embarks upon the endeavor to focalize meticulously upon the mechanistic sway exerted by the enteric microbiota upon AD, plunging profoundly into the execution of interventions that govern the milieu of enteric microorganisms. In doing so, it bestows relevance upon the therapeutic stratagems that form the bedrock of AD’s management, all whilst casting a prospective gaze into the horizon of medical advancements.

## 1. Introduction

Alzheimer’s disease (AD) represents a progressive neurodegenerative disorder characterized by the degeneration of cognitive domains, encompassing but not confined to memory, language, visual–spatial function, and executive abilities. The transition of AD patients unfurls from initial phases typified by apathy and depression to subsequent stages distinguished by communication difficulties, orientation disturbances, cognitive disarray, and impaired judgment, potentially impacting one or multiple cognitive domains (D'Onofrio et al., 2012). Epidemiological investigations have highlighted AD as the most prevalent neurodegenerative disorder beleaguering the geriatric populace on a global scale. Within the last two decades, the mortality rate associated with AD has undergone a marked escalation, eclipsing a staggering 145% (Jia et al., 2020; Alzheimer’s disease facts and figures, 2023). In the current research milieu, conjectures proffer that the neuropathological signatures of AD encompass the emergence of amyloid-beta (Aβ) neuritic plaques, the presence of hyperphosphorylated tau protein within neurofibrillary tangles (NFTs), in conjunction with the attrition and debilitation of neuronal constituencies and synaptic junctions (Long and Holtzman, 2019).

In recent times, substantial attention has been directed towards the elucidation of the implication of the gut microbiota in AD. The gut microbiota denotes a complex assemblage of diverse microorganisms inhabiting the human digestive tract. This intricate ecosystem comprises bacteria, fungi, viruses, archaea, and protozoa, distinguished by their morphological, physiological, and genetic attributes (Whitman et al., 1998; Zhang et al., 2021). A mounting body of experimental and clinical evidence indicates that the gut microbiota holds the potential to exert influence on the brain through the multifaceted gut-brain axis, encompassing the immune, metabolic, endocrine, and nervous systems, as well as the gut-brain barrier (Guarner and Malagelada, 2003; Adak and Khan, 2019). Consequently, perturbations in gut ecology and the labyrinthine interplay between microbiota and the host manifest as pivotal contributory agents in the milieu of AD.

In the practical realm of pharmaceutical application, in comparison to agents that exert their effects upon the cerebral domain, medications acting upon the intestinal microbiota possess the distinctive capacity for directly modulating the composition and functionality of the gut microorganisms, obviating the necessity of traversing the blood–brain barrier to gain access to the cerebral milieu. On the axis of safety, methodologies centered around modulating the gut microbiota are conventionally deemed to embody a relative sense of security. Pertaining to therapeutic efficacy, the orchestration of the gut microbiota stands poised to systematically ameliorate the overall somatic well-being. Within the expanse of this comprehensive survey, we shall embark upon a discourse concerning the manner by which the intestinal microbial consortium establishes an intricate intersection with the cerebral domain, elucidating its mechanistic involvement in the genesis and progression of AD. Our focal point shall revolve around the contemplation of the contemporary landscape and potentiality inherent in the alteration of the intestinal microbial milieu and its consequential metabolic fluctuations, in the context of avenues for amelioration and treatment vis-à-vis the realm of AD pathology.

## 2. Intestinal microbiota

With the advent of high-throughput sequencing techniques, our comprehension of the extraordinary microbial community residing within the human gut has deepened. The population of the gut microbiota is extensive, and its diversity and abundance are host-specific, subject to various influences such as gender, age, dietary nutrition, and geographic environment (Backhed et al., 2005; Rajilic-Stojanovic et al., 2007; Hugon et al., 2015; Johnson et al., 2019). The gut microbiota thrives within the surfaces and fluids (external symbionts) of multicellular organisms, encompassing the skin, digestive tract, and respiratory tract. Within the confines of the digestive tract alone, an estimated 10^14^ distinct species of gut microbiota exist (Whitman et al., 1998; Guarner and Malagelada, 2003). As expounded within the genetic reservoir of the human gastrointestinal microbiome, a myriad of symbiotic bacteria and archaea, engaged in mutually advantageous relationships, collectively constitute an expansive chemical forge characterized by inherent dynamism. These entities possess the inherent capability to biosynthesize a diverse array of compounds vital for their own metabolic maintenance, concurrently coordinating the assembly of molecules that resonate throughout the expanse of the biotic domain. The interplay with the host’s physiological well-being remains profoundly interwoven; however, the intricate biological tableau orchestrating within the human organism, coupled with the intricate interplay of multifarious factors, presents a formidable challenge when attempting to distill the impact of the microbial consortium on the central nervous system. The precise operative mechanisms continue to linger cloaked in ambiguity. Consequently, this domain is beset by a myriad of challenges, compelling a deeper investigative pursuit to meticulously unravel the intricate interrelation between the microbiota and the central nervous system, alongside their precise contributions to diverse pathological conditions and states of health.

### 2.1. Classification of intestinal microbiota

Prior to birth, during the prenatal period, the human intestinal tract exists in a microorganism-free state within the confines of the mother’s womb. However, following birth, the colonization of the gut microbiota rapidly initiates through contact with the maternal birth canal and exposure to external environmental factors (Chong et al., 2018). As time progresses, both the diversity and abundance of the gut microbiota increase progressively in conjunction with age. The classification of gut microbiota is typically based on ecological characteristics, morphology, and physiological traits, with taxonomic classification at the bacterial genus level being the most commonly employed method. Based on current research in human microbiology, the intestinal microbiota within the human body predominantly comprises the phyla *Firmicutes*, *Bacteroidetes*, *Proteobacteria*, and *Actinobacteria*. Furthermore, the composition of microbial communities within the intestinal tracts of distinct individuals exhibits variation, and their respective functionalities are also unique (Hayashi et al., 2002; Hold et al., 2002; Wang X. et al., 2003; Eckburg et al., 2005; Arumugam et al., 2011; Hugon et al., 2015).

The *Firmicutes phylum* stands as a prevailing consortium of microorganisms within the human gastrointestinal tract, embracing a quintet of genera, to wit: *Clostridium*, *Ruminococcus*, *Faecalibacterium*, *Eubacterium*, and *Lactobacillus* (Hayashi et al., 2002; Eckburg et al., 2005; Wall et al., 2007). Emerging as the subsequent salient cohort within the human intestinal milieu, the *Bacteroidetes phylum* embodies Gram-negative obligate anaerobes. Drawing upon insights extrapolated from the evolutionary lineages discerned via 16S rRNA gene phylogenetics, it partitions into three principal clades: *Bacteroides*, *Prevotella*, and *Porphyromonas* (Salyers, 1984; Patrick, 2015; Wang C. et al., 2021). Operating predominantly as facultative anaerobes, the *Proteobacteria phylum* contends with the exacting demands imposed by the anaerobic milieu of the intestinal tract, within which a substantial contingent therein undertakes a pathogenic vocation (Lupp et al., 2007). Conversely, within the *Actinobacteria phylum*, a cohort characterized as Gram-positive obligate anaerobes, a nuanced yet pivotal presence emerges. Although not wielding numerical dominance in the intricate milieu of the intestines, it is within this cohort that the genus *Bifidobacterium* finds its abode. Noteworthy is the prevalence commanded by this genus within the confines of the human intestinal milieu, where it assumes a role of pronounced significance as a purveyor of probiotic influence, concomitantly contributing to the overarching landscape of human health (Cani et al., 2007; Shkoporov et al., 2008; Barka et al., 2016). While certain assemblages of bacterial constituents may harbor proclivities towards pathogenic proclamations, as evidenced by their propensity to incite inflammatory retorts, compromise intestinal epithelial fortifications, and incite orchestrated immune rejoinders, it is paramount to recognize that a substantive faction of the intricate intestinal microbiota is deeply embroiled in multifarious metabolic enterprises. These encompass an expansive portfolio including but not limited to polysaccharide catabolism, intricate conversions of bile acids and steroids, endogenous synthesis of vital vitamins, intricate biotransformations of polyphenolic entities, orchestration of immune modulatory responses, and the efficacious expulsion of entrenched pathogens. Collectively, these orchestrated endeavors undertake a momentous mantle, profoundly interfacing with facets of human nourishment, foundational metabolic orchestration, and immunological processes of marked consequence (Falagas and Siakavellas, 2000; Loubinoux et al., 2002; Casterline et al., 2017). It is through these intricate symphonies of activity that a pivotal foundation is laid, one that fundamentally upholds the delicate equilibrium underpinning the tapestry of human physiological well-being.

## 3. Alzheimer’s disease

AD stands as a multifaceted neurodegenerative disorder marked by gradual cognitive decline, encompassing various pathological factors. The etiological underpinning of this affliction intricately intertwines with the anomalous aggregation of Aβ peptides within aged plaques, forming a convoluted presentation. This occurrence arises as a resultant consequence of enzymatic cleavage endured by the amyloid precursor protein (APP) along its proteolytic trajectory. Moreover, AD correlates with the decline of cholinergic neurons and the onset of neurofibrillary tangles provoked by hyperphosphorylated tau (Knopman et al., 2021). Amid the diverse array of theories expounding on the pathogenesis of AD, the present chapter predominantly directs its focus toward the overarching mechanisms that substantiate AD pathogenesis, in conjunction with potential pathways associated with the gut microbiota.

### 3.1. The amyloid cascade hypothesis

The amyloid cascade hypothesis retains its dominion as the preeminent postulate pertaining to the etiological substrates of AD. This proposition proffers that the neurodegenerative mechanisms within the ambit of AD find their chief impetus in the aberrant genesis and conglomeration of Aβ proteins, inciting neuronal perturbation and attenuated synaptic malleability (Selkoe, 1991; Selkoe and Hardy, 2016). The quintessential moieties constituting the senile plaques that materialize within the AD cerebral milieu predominantly enshroud Aβ, originating from the proteolytic cleavage of the amyloid precursor protein (APP) through discrete secretase moieties (β- and γ-secretases; Soldano and Hassan, 2014).

Aβ, a peptide, exhibits various variants, with Aβ40 playing both toxic and protective roles, while Aβ42 demonstrating higher neurotoxicity compared to Aβ40 (Yankner et al., 1990; Murray et al., 2009). Mutations in genes such as APP, PSEN1, and PSEN2 can lead to the production of longer and more abundant Aβ species. In terms of Aβ clearance mechanisms, genetic variations of ApoE4 and impaired Aβ degradation can contribute to the accumulation and oligomerization of Aβ in the peripheral and association cortices. Aβ oligomers can impact synapses, contributing to the formation of NFTs that gradually diffuse into plaques (Szaruga et al., 2017). Additionally, Aβ42 oligomers can activate microglia and astrocytes, triggering an inflammatory response that disrupts neuronal ion homeostasis and results in oxidative damage (Hardy and Selkoe, 2002; Selkoe and Hardy, 2016).

The accumulation of Aβ gives rise to synaptic dysfunction, impairments in dendritic spine and synaptic plasticity, as well as defects in neurotransmitter systems, ultimately leading to cognitive impairments. Even in the absence of plaques, Aβ exerts synaptic toxicity and has a dual effect on synaptic function. At physiological concentrations, Aβ enhances N-methyl-D-aspartate receptor (NMDAR) excitation, promoting presynaptic functions and enhancing synaptic release. However, at pathological concentrations, Aβ reduces presynaptic efficacy. Abnormally elevated levels of Aβ42 show a strong affinity for GluN2B-containing NMDARs, disrupting synaptic plasticity, and resulting in postsynaptic inhibition and loss of dendritic spines (Mucke et al., 2000; Mucke and Selkoe, 2012; Dupuis et al., 2023). Furthermore, Aβ triggers intermittent neuronal hyperactivity in the cortex and hippocampus prior to plaque formation, leading to significant remodeling of inhibitory neural circuits and excessive inhibition of granule cells. This creates a cycle of sustained neuronal overactivation (Palop et al., 2007; Zott et al., 2019). Collectively, these processes impair the structure and function of neurons, ultimately contributing to the development of AD.

### 3.2. Glial cells

Recent research has revealed that alterations in microglia and astrocytes contribute to the latent progression of AD prior to the onset of cognitive impairments (De Strooper and Karran, 2016). Microglia, the resident immune cells in the brain, continuously monitor the microenvironment under normal physiological conditions. In AD patients, abnormalities in neuroglial cells (microglia, astrocytes, and neurons) result in the production of pro-inflammatory cytokines (such as interleukin-1β [IL-1β], IL-6, tumor necrosis factor-alpha [TNF-α], etc.), chemokines, the complement system, as well as reactive oxygen and nitrogen species. These factors disrupt neuronal activity at nerve terminals, leading to synaptic dysfunction and loss, which is associated with memory decline (Lawson et al., 1990; Orsini et al., 2014; Wang et al., 2015). However, activated microglia, apart from their pro-inflammatory role, can also release anti-inflammatory factors (such as IL-10, arginase-1, etc.) to repair damaged neural tissue, exhibiting an anti-inflammatory phenotype. The detailed mechanisms underlying the dual role of M1/M2 microglia in AD are still not fully understood (Subhramanyam et al., 2019; Wang Q. et al., 2021).

Astrocytes, on the other hand, play a crucial role in modulating neuronal and vascular functions. They can regulate the properties of the BBB through specific signaling mechanisms in their endfeet. Disruption of astrocytic endfeet can lead to BBB leakage, compromising its integrity (Acosta et al., 2017).

### 3.3. Glucose metabolism

The metabolic processes related to glucose, including insulin signaling transduction and glucose metabolism, play a significant role in the pathophysiology of AD and neuronal senescence. The reduced uptake of glucose in critical brain regions, leading to inadequate energy supply for neurons, is associated with cognitive decline observed in AD patients (Kim and Egan, 2008; Wang Q. et al., 2022). Insulin and incretins, such as glucagon-like peptide 1 (GLP-1) and glucose-dependent insulinotropic polypeptide (GIP), are involved in the regulation of glucose homeostasis and have complex effects on neurodegenerative processes.

Insulin traverses the intricate barricade of the BBB through specialized insulin transporters, thereby exerting its modulatory prowess upon ubiquitous insulin receptors, ubiquitously scattered across the expanse of the cerebral terrain. These receptors find their abode nestled amidst the intricate network of neurons and glial cells, assuming a cardinal mantle in the orchestration of cellular metabolic cascades, neuronal ontogenesis, and divergence, transcriptional orchestration of genetic blueprints, plasticity of synapses, and preservation of neural architecture (Blazquez et al., 2014; Rhea et al., 2018; Shaughness et al., 2020).

Perturbation in insulin functionality, deemed insulin resistance, manifests as a decrement in the receptivity of designated tissues to the orchestrated maneuvers of insulin, thus engendering a plausible nexus with the intricate choreography of typical AD hallmarks, namely the orchestrated aggregation of Aβ and tau proteins (Pivovarova et al., 2016; Akhtar and Sah, 2020; Nowell et al., 2023). Scrutinizing endeavours have unveiled that peptides resembling the glucagon-like ilk, notably GLP-1 and GIP, rouse the entero-insulin signaling axis into motion, thus assuming the custodianship of glycemic equilibrium. Operating through a plenitude of mechanisms, encompassing the tempering of neural inflammatory surges, modulation of tau phosphorylation gradients, augmentation of synaptic efficacy, mitigation of amyloidogenic protein agglomerations, and abatement of insulin resistance, these agents wield a profound sway over neuronal kinetics, consequently casting a benevolent cadence upon mnemonic capacities (Grieco et al., 2019). In murine paradigms, the involvement of GLP-1 in fortifying the bedrock of learning and memory (During et al., 2003; Isacson et al., 2011), embellishing the bulwarks of neuroprotection and synaptic plasticity within the hippocampal precincts (McClean et al., 2011; Porter et al., 2011), whilst concurrently reining in the specter of β-amyloid plaque accretions and activation of microglial cohorts in models mimicking the facets of AD, has been duly showcased (McClean et al., 2011). Notwithstanding these advancements, the temporal trajectory underpinning the emergence of insulin resistance within the precincts of AD patients remains an enigma, enshrouded in the intricacies of its multifaceted manifestation. The etching of aberrant insulin signaling onto the canvas of AD appears to be an intricate tapestry, intricately interwoven within the labyrinthine recesses of the ailment’s pathogenesis (Ghasemi et al., 2013).

### 3.4. Blood–brain barrier

BBB comprises an endothelial cell-based membranous lining situated within cerebral micro-vessels. This intricate structure functions as a pivotal interface, meticulously orchestrating interactions among the immune system, neural cells, and circulatory entities (Sagare et al., 2012).

In the realm of AD, the intricate interplay of molecular events involving alterations and malfunctions of the BBB has garnered substantial attention due to its profound implications for the initiation and advancement of chronic inflammatory processes. Both tau protein and Aβ have been distinctly implicated in the perturbation of BBB integrity, thereby instigating a detrimental cascade that amplifies the course of neurodegenerative processes and the consequent inflammatory responses. The deposition of Aβ within the vascular framework triggers pro-inflammatory and cytotoxic cascades, leading to increased permeability of the BBB in AD patients (Zenaro et al., 2017). The emergence of tau protein in the perivascular vessels encircling the hippocampal terrain exhibits a noteworthy correlation with the compromised state of BBB functionality. These fissures in the robustness of BBB integrity bestow passage to molecules of an inflammatory nature and immune effectors, thereby augmenting the overall inflammatory milieu characterizing AD (Blair et al., 2015; Cai et al., 2018). Preserving the function and integrity of the BBB is emerging as an important aspect in the context of AD. Strategies aimed at modulating BBB function and reducing its permeability may have therapeutic potential in mitigating neuroinflammation and limiting the progression of AD. Consequently, the modulation of BBB function and the preservation of its integrity emerge as pivotal considerations within the realm of AD investigation. Notably, the imperative quest persists for augmented investigations that are poised to unravel the intricacies enshrouding BBB dysfunction in the context of AD, consequently furnishing targeted modalities for the preservation of BBB integrity in this pathological milieu.

### 3.5. Other pathological mechanisms

Beyond the elucidated pathological pathways, a multitude of factors converge in shaping the trajectory of AD development. These factors encompass diverse aspects, including post-translational modifications of tau protein, namely phosphorylation, truncation, and glycosylation, alongside impairments in the autophagy-lysosomal pathway, mitochondrial dysfunction, aberrant cholinergic transmission, oxidative stress, genetic susceptibility, aging, and influences stemming from lifestyle and environmental factors (Guo et al., 2020).

## 4. Intestinal microbiota and AD

### 4.1. Alterations in intestinal microbiota in AD

Numerous inquiries have unveiled substantial perturbations in both the abundance and composition of the intestinal microbiota among individuals afflicted with AD, as well as within corresponding animal models. These alterations encompass a discernible reduction in the diversity of gut microbiota and the prevalence of probiotic strains, concomitant with an elevation in pro-inflammatory bacterial populations and their derivatives. These shifts are intricately intertwined with the underlying pathogenesis of AD. A preceding investigation notably underscored that prior to the detection of cerebral Aβaggregation, a conspicuous disruption in the homeostasis of the intestinal milieu becomes manifest in the Tg2576AD murine model (Honarpisheh et al., 2020). A rigorous analysis employing high-throughput 16S rRNA sequencing of fecal specimens collected from AD patients, individuals with Mild Cognitive Impairment (MCI), and healthy counterparts has brought to light a noteworthy reduction in the abundance of microbial species responsible for the production of short-chain fatty acids within the AD cohort, when juxtaposed with normative subjects (Verhaar et al., 2021). Empirical evidence supports the assertion that within the AD spectrum, taxa such as *Bacteroidetes*, *Fusobacteria*, *Bifidobacterium*, and *Lactobacillus* exhibit conspicuously heightened abundance, in stark contrast to the health control group. Conversely, the prevalence of *Firmicutes* and *Clostridiaceae* is distinctly subdued in the AD cohort. Furthermore, these observed variations in microbial abundance follow a discernible gradient of alteration from the early stages of MCI to the more advanced phases of AD progression (Vogt et al., 2017; Hung et al., 2022).

An additional investigation, delving into the intricate interplay between specific bacterial taxa and cerebral amyloidosis within individuals afflicted by cognitive impairment, unveils a conspicuous nexus. The deposition of cerebral amyloid in patients with cognitive impairment exhibits a concordance with heightened fecal concentrations of pro-inflammatory taxa, exemplified by *Escherichia* and *Shigella*, juxtaposed with the waning presence of anti-inflammatory taxa typified by rectal *Bacteroides* and *fragile Bifidobacterium* (Cattaneo et al., 2017). Subsequent inquiries not only elucidate an inverse correlation between amyloidogenic protein content and the abundance of fecal lactobacilli in AD patients but also establish a positive correlation with lipopolysaccharides and Gram-negative colonic *Enterobacteriaceae* (Li et al., 2019). In a pioneering endeavor, Dodiya et al. (2019) performed fecal microbiota transplantation from AD mice into therapeutically treated AD mice, resulting in the restoration of dysbiotic gut microbiota and partial recovery in Aβ pathology and microglial morphology. This substantiates the causal role of the microbiota in regulating Aβ and microglial physiology within the AD murine model.

### 4.2. Modulation of AD progression by the gut microbiota

The gut-brain axis delineates a foundational biological framework expounding the dynamic interplay underpinning the gastrointestinal tract and the cerebral domain. This intricate system orchestrates a myriad of visceral organs and intricate tissues, encompassing the expansive intestinal milieu, neural networks, and the immunological infrastructure (Strati et al., 2017). These elements engage in elaborate neural and chemical dialogues, fostering intricate interchanges. The symbiotic symphony of this interaction assumes paramount import within arenas of affect, behavior, and cognition. The composite constituents constituting the enteric-cerebral axis encompass these pivotal anatomical entities: (i) The alimentary canal, pivotal not solely in digesting and absorbing nutrients but also as a thriving niche for a profusion of symbiotic microorganisms. (ii) The enteric nervous system, often colloquially referenced as the “second neuronal center,” exercises autonomic dominion over gastrointestinal motility and secretory functions, engaging in reciprocal discourse with the cerebral realm via neurotransmitter-mediated transmissions. (iii) The central nervous system, epitomizing the body’s preeminent command nexus, harmonizes bidirectional influence with the intestinal milieu and the enteric nervous network through intricate neurotransmitter signaling. (iv) The immune ensemble, paramount in fortifying the organism against pathogens and maintaining immune equilibrium, expedites signal transduction linking the enteric milieu and the cranial expanse through specialized immune effectors and mediators. (v) The microbial consortium, pivotal in digestive processes, immune modulation, and neural conductions, assumes an indispensable mantle.

**Figure 1:**
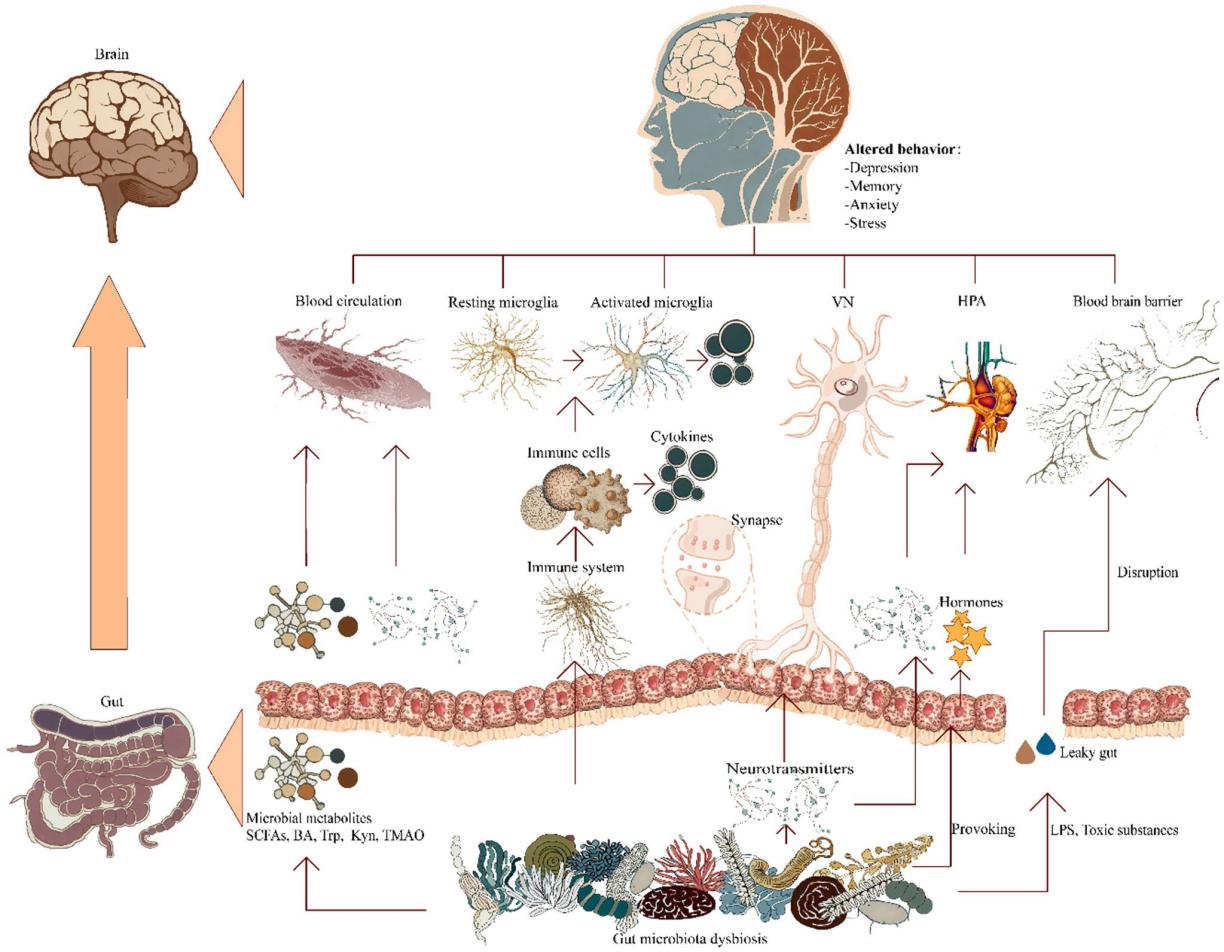
Ramifications of gut microbiota dysbiosis on cerebral pathways. A pictorial representation elucidating the manifold established conduits of communication within the gastrointestinal-neuronal axis in relation to the gut microbiota associated with AD, encompassing: (i) microbial metabolic pathways; (ii) immune regulatory pathways; (iii) vagus nerve’s neurologic circuits; (iv) signaling cascades governing neuroendocrine interplay; and (v) provocation of blood–brain barrier compromise. BA, bile acids; HPA, hypothalamic–pituitary–adrenal; Kyn, kynurenine; LPS, lipopolysaccharide; SCFAs, short-chain fatty acids; TMAO, trimethylamine-N-oxide; Trp, tryptophan; VN, vagus nerve.

At present juncture, it is widely accepted that the modalities governing interconnections and signaling amongst these anatomical entities are principally channeled through five discernible communicative pathways interfacing with the central nervous system: the gut microbiota metabolic pathways, the immunological itinerary, the vagal neural trajectory, the neuroendocrine avenue, and the modulation of the BBB (Figure 1). These pathways may act in autonomy or synergistically, thus intricately molding the etiology and progression of AD (Carabotti et al., 2015; Agirman and Hsiao, 2021; Ding et al., 2021).

#### 4.2.1. Gut microbiota and metabolism

The gut microbiota possesses the capacity to engage, whether by direct action or indirect mediation, in the production of diverse metabolites. This repertoire spans neurotoxic agents, short-chain fatty acids, amino acid derivatives, bile acids, trimethylamine-based compounds, as well as lipopolysaccharides (LPS; Kowalski and Mulak, 2019). Through such multifaceted biochemical contributions, the gut microbiota consequently wields a notable impact upon the operational dynamics and comportment of the central nervous system.

Short-chain fatty acids (SCFAs), including butyrate, propionate, and acetate among others, as the resultant products of dietary fiber fermentation, intricately governed by the gastrointestinal microbiota, primarily associated with the *Bacteroidetes phylum* (Koh et al., 2016). The study unveiled that SCFAs across the formidable BBB via monocarboxylic acid transporters localized upon the endothelial milieu (Hoyles et al., 2018). Remarkably, SCFAs demonstrate the potential to maintain the integrity of the BBB, hinder diverse pathways that connect non-specific inflammatory responses of the BBB to extracorporeal microbial infections, and moreover, foster the reinstatement of BBB permeability in murine models simulating cerebral injuries (Braniste et al., 2014; Li et al., 2016). Furthermore, SCFAs might wield an influence upon the peripheral immune milieu, orchestrating the modulation of cerebral functionality. By virtue of enhancing the intestinal barrier and intercepting the translocation of bacteria and bacterial constituents, or through direct reciprocal interplays microglia, thereby impacting their morphological attributes and operational dynamics, these compounds transmute their capability to sequester antigens and alleviate the synthesis of proinflammatory cytokines such as IL-12 and TNF-α, thus indirectly assuaging systemic inflammation (Chang et al., 2014; Corrêa-Oliveira et al., 2016; Sun J. et al., 2020). This cascading effect could potentially yield a diminution in neuroinflammation and the accrual of Aβ in the cerebral milieu. The orchestration of gene expression hinges upon the manipulation of chromatin conformation encompassing DNA, primarily executed via histone acetylation (Marks et al., 2004). Histone deacetylases (HDACs) emerge as pivotal arbiters in cerebral ontogeny, as well as a spectrum of neuro-psychiatric maladies, spanning but not confined to depression, schizophrenia, and AD (Volmar and Wahlestedt, 2015). Indications underscore that SCFAs also manifest ameliorative impacts on erudition and memory in specimens of the wild type and neurodegenerative murine prototypes (Fischer et al., 2007). This facilitation stems from HDAC inhibition mediated by SCFAs, coupled with the modulation of neurotrophic agents (Stafford et al., 2012).

While being endogenously generated by the host’s hepatic system, bile acids (BA) serve a purpose within the gastrointestinal tract, facilitating the digestion and absorption of lipids. Nevertheless, the intricate milieu of the intestinal microbiota harbors the inherent capability to metabolize BA, thereby intricately engaging in the enterohepatic circulation and intricate processes of cholesterol metabolism. The intricate metabolism and biotransformation of BA inherently demand the dynamic engagement of the intestinal microbiome (Winston and Theriot, 2020). Supported by both empirical and clinical evidence, the proposition gains ground that cerebral specimens afflicted with AD evince perturbations in BA signaling in contrast to their cognitively intact counterparts (Mahmoudiandehkordi et al., 2019). These deviations materialize as perturbed BA concentrations not only in cerebrospinal fluid and serum but also as anomalies in cholesterol metabolism (Baloni et al., 2020). Consequently, a compelling deduction emerges, postulating that the microbiota might wield a discernible influence over the BA-mediated repercussions associated with AD. The precise mechanics undergirding this intricate interplay ostensibly intertwine with the disruptive potential inherent to anomalous bile acid species, thereby culminating in an exacerbated permeability of the cerebral BBB (Quinn et al., 2014). Intriguingly, among these species, Taurochenodeoxycholic acid, an indigenous hydrophilic bile acid, emerges as a pivotal protagonist, effectively modulating cerebral Aβ levels through its adept manipulation of lipidomic metabolic trajectories (Nunes et al., 2012).

Metabolites originating from the gut microbiota assume a pivotal role in orchestrating tryptophan (Trp) metabolism within the intricate landscape of AD, encompassing intricate pathways involving ligands for the aromatic hydrocarbon receptor, the kynurenine (Kyn) pathway, and the serotonin pathway (Hartai et al., 2007; Giil et al., 2017). Both Trp and Kyn demonstrate the ability to traverse the formidable blood–brain barrier. Subsequent metabolites, including quinolinic acid, wield a notable impact on neurotransmitter metabolism through modulation of the NMDAR, thus exerting a discernible influence on cognitive faculties within the cerebral domain (Agus et al., 2018). Furthermore, exploratory pursuits propose that the introduction of tryptophan metabolic derivatives like indole or bacterial tryptophanase could potentially choreograph regulatory mechanisms governing astrocytic function. This, in turn, holds the promise of mitigating inflammation within the central nervous system among cohorts of murine subjects (Rothhammer et al., 2016).

The gut microbiota exhibits a distinctive capability for the bioconversion of dietary moieties containing methylamine, giving rise to trimethylamine, subsequently subject to hepatic flavin monooxygenase-mediated transformation into trimethylamine N-oxide (TMAO; Tang et al., 2013; Wang et al., 2014). Recent investigations have brought to light an inverse correlation between levels of TMAO and cognitive acuity, potentially linked to hippocampal inflammation and the genesis of reactive oxygen species. Experiments involving the administration of TMAO to aged mice have evidenced heightened microglial activation and escalated inflammatory mediators (Meng et al., 2019; Brunt et al., 2021).

In a parallel vein, specific bacterial strains, typified by cyanobacteria, lay claim to the ability to synthesize neurotoxic agents, inadvertently integrated into peptide sequences within the cerebral domain, thus setting the stage for protein misfolding and the ensuing genesis of amyloid plaques that epitomize the AD phenotype. This intricate interplay ultimately lends itself to the disruption of neurological homeostasis and functions (Szablewski, 2018).

#### 4.2.2. Immune pathways

The intricate and meticulously organized nature of both the immune system and the central nervous system is evident as they choreograph and oversee a myriad of physiological functions. Their operative frameworks and developmental paths showcase shared characteristics that may be intricately interwoven with the genesis of neuropsychiatric ailments (Capuron and Miller, 2011; Miller and Raison, 2016). Within the cohort afflicted by AD, the deposition of cerebral Aβ could potentially incite immune-inflammatory retorts by engaging Toll-like receptors (TLRs) and effectuating CD14 activation—primarily orchestrated by microglial cells (Erny and Prinz, 2020; Sorboni et al., 2022). This symphony culminates in the exudation of a spectrum of cytokines and the induction of a diverse repertoire of antigenic markers, thereby kindling neuroinflammatory responses. This acute, self-limited inflammatory reaction performs the dual role of expediting Aβ clearance and reinforcing neuronal fortification. The potential disruption of pro-inflammatory gut microbiota in AD patients might conceivably act as a catalyst, fostering the aggregation and configuration of Aβ proteins driven by inflammation (Sorboni et al., 2022). Currently, the gastrointestinal microbiome’s substantial capacity to influence the maturation of immune cells has been elucidated, positioning it as a pivotal participant in the developmental trajectory of cerebral immunity.

The potential dysbiosis of pro-inflammatory gut microbiota in individuals with AD has the potential to trigger inflammation, thus fostering the genesis and amalgamation of Aβ proteins. Evidentiary groundwork has firmly established that the gastrointestinal microbiota can influence the development of immune cells and play a central role in the immunomodulation of the brain (Cattaneo et al., 2017). Alterations in the composition of the gut microbiota and the metabolites it produces can regulate immune responses in various cell types, depending on the immune environment. Microbiota-derived metabolites such as taurine, histamine, indole, and spermine collectively modulate the secretion of NLRP6 inflammasome, IL-10, and IL-18, thereby impacting downstream bioactive peptides and correlating with the levels of inflammatory factors and the severity of AD (Elinav et al., 2011; Levy et al., 2015; Alexeev et al., 2018). Furthermore, the gut microbiota has the ability to dispatch signals to the brain through its influence on peripheral immune cells. Within this purview, SCFAs emerge as linchpins, adroitly choreographing immune modulations across diverse cellular phyla inclusive of colonocytes, neutrophils, and T lymphocytes. An aptitude to foment and modulate T-cell differentiation pathways — steering them along the trajectories of T helper 1 (Th1) and Th17 archetypes, alongside interleukin-10-equipped regulatory T cells — is among the repertory of SCFA effects unveiled. These effects of SCFAs contribute to the promotion of immune responses or immune tolerance, contingent upon the contextual setting (Furusawa et al., 2013; Park et al., 2015; Liu et al., 2020).

Serum amyloid A (SAA) emerges as a prominent acute-phase protein intricately associated with the intricate interplay between gut microbial ecology and inflammation. In the context of murine models manifesting AD pathology, astute observation reveals the focal instantiation of SAA, existing in harmonious concert with senile plaques. By inciting the differentiation of Th17 cells, SAA orchestrates the amplification of pro-inflammatory cytokines, exemplified by IL-17 (a potent instigator of cytokines such as IL-1β, IL-6, TNF-α), along with the augmentation of IL-22 (Zhang et al., 2013; Lee J. Y. et al., 2020). Thus, it functions as a conduit facilitating neuroinflammation and activation of glial cells, collectively exerting their sway upon the panorama of AD. The systemic inflammation borne from the dysregulation of the intestinal milieu culminates in undue hyperactivation of microglia and derangement of hippocampal plasticity, thereby further fueling the inception and progression of AD (Saiyasit et al., 2020).

#### 4.2.3. The vagus nerve pathway

The vagus nerve (VN), encompassing a composition of 80% afferent fibers and 20% efferent fibers, exercises authoritative dominion over virtually the entirety of the gastrointestinal tract. It presents itself as a neural entity of intricate complexity and pivotal significance, wielding an expansive scope of influence that encompass diverse dimensions of organismal physiology, spanning sensory, motor, and autonomic functions (Han et al., 2022). As a principal constituent within the autonomic nervous system, the VN mastermind bidirectional conduits of communication with visceral organs, thus superintending organ functionality and preserving organismal internal homeostasis (Bonaz et al., 2017). Despite the indirect engagement of the VN’s afferent pathways with the gastrointestinal microbiota or luminal contents, these pathways nonetheless possess the intrinsic capability to discern luminal cues via a process of diffusion across the gastrointestinal barrier, a phenomenon facilitated by bioactive bacterial compounds or metabolites, such as serotonin and enteric gut hormones (Li et al., 2000; Chaudhri et al., 2006). By eliciting electrical stimulation of the afferent fibers comprising the VN, a potential for modulation of neurotransmitter levels within the cerebral realm emerges (Ressler and Mayberg, 2007). Moreover, select bacterial strains and byproducts, exemplified by LPS (Hosoi et al., 2005), similarly harbor the latent capacity to indirectly incite afferent fibers within the VN, thereby effectuating a discernible impact upon cerebral functionality.

Enteroendocrine cells nestled within the intricate confines of the intestinal tract establish intimate connections with the afferent fibers of the VN, thereby engendering a direct line of neural dialogue (Han et al., 2022). This sensory conduit, in its turn, assumes the mantle of an information emissary, transmitting its cargo to the central autonomic network for intricate analysis and seamless integration. This intricate lattice of interconnected nodes encompasses structures of discernable import, including but not limited to, the parabrachial nucleus, the locus coeruleus, the hypothalamus, and the limbic system, wherein the thalamus, amygdala, and hippocampus find their residence (Schroeder and Backhed, 2016; Han et al., 2022). Empirical inquiry has laid bare a noteworthy revelation: administering chronic VN stimulation to AD rodents exerts a discernibly positive influence upon their cognitive faculties. Plausible attribution for this cognitive amelioration may be accorded to the modulation of glutamatergic receptor quantities by the agency of the VN (Yesiltepe et al., 2022). Notably, the act of vagal stimulation serves as the catalyst for the activation of specific neuronal enclaves, colloquially denominated as “blue spots.” This activation, in a cascading fashion, precipitates the liberation of catecholamines within strategic redoubts like the hippocampus and neocortex, thereby engendering a milieu conducive to the potentiation of synaptic plasticity while concurrently reining in the surges of inflammatory signaling (Vargas-Caballero et al., 2022).

Beyond its hitherto expounded role as an intermediary governing the exchange of neural missives betwixt the enteric expanse and the central nervous system, the VN emerges as a conduit of unexpected import, affording direct bacterial signaling an unforeseen passage to the inner sanctum of the brain. Lee K. E. et al. (2020) successfully isolated *Paenalcaligenes hominis* from fecal samples obtained from both elderly humans and aged mice. The subsequent transplantation of this microorganism into juvenile mice yielded intriguing results. Specifically, their investigations revealed the profound impact of *P. homanis* on hippocampal functionality, consequently leading to a notable decline in cognitive capabilities. This deleterious effect was attributed to the actions of extracellular vesicles. Notably, the implementation of vagal denervation surgery exhibited a remarkable efficacy in ameliorating the cognitive deficits induced by *P. homanis*. Moreover, this surgical intervention demonstrated an additional benefit by preventing the infiltration of extracellular vesicles into the hippocampal region (Lee K. E. et al., 2020). Furthermore, emerging evidence underscores the capacity of vagal nerve (VN) fibers to intrinsically synthesize and release acetylcholine (ACh), thereby eliciting a discernible impact upon cholinergic neurons, a phenomenon that has not escaped scholarly attention. Particularly noteworthy is the conspicuous reduction in cholinergic neuronal contingent observed among those afflicted by the scourge of AD. Through assiduous inquiry, it has been ascertained that the ACh liberated consequent to the excitation of VN efferent fibers assumes a quelling role in the orchestration of TNF-α secretion. This regulatory effect unfolds through a complex interplay wherein the α-7 nicotinic ACh receptor, resident on macrophagic substrates, engenders interaction with the aforementioned ACh, culminating in a cascade yielding a discernibly anti-inflammatory demeanor (Wang H. et al., 2003).

While the salient involvement of the VN within the milieu of the gastrointestinal domain stands as an unequivocal tenet, the intricate conduit governing its functional purview, replete with its labyrinthine nuances, is progressively unfurling its enigma. As we navigate this scientific terrain, the import of the VN assumes reverberating significance vis-à-vis the intricate tapestry of the gut microbiota, behavioral dynamics, and the inexorable progression of neurodegenerative afflictions. Yet, in the midst of this irrefutable import, it becomes incumbent upon the academic community to underscore the imperative for sustained scholarly forays, wherein validation and elucidation of its cardinal role in orchestrating the interplay and paradigmatic shifts underpinning the realm of intestinal microflora and the onset of neurodegenerative pathologies are pursued with unwavering vigor.

#### 4.2.4. The neuroendocrine system

Certain microorganisms manifest the capability to biosynthesize neurotransmitters, such as γ-aminobutyric acid (GABA), taurine, and 5-hydroxytryptamine (5-HT). These bioactive compounds possess the inherent potential to exert modulatory effects on neural transmission within the confines of the central nervous system. The metabolic perturbations orchestrated by these microorganisms carry the capacity to give rise to profound ramifications for individuals grappling with the complexities of AD.

Empirical investigations have revealed that disturbances within the compositional fabric of the microbial consortium precipitate the accrual of peripheral phenylalanine and isoleucine, as delineated in references (Griffin and Bradshaw, 2017; Fujii et al., 2019; Wang X. et al., 2019). These molecular perturbations, in a cascade fashion, function as pivotal agents in fomenting the inception of neuroinflammatory cascades, displaying a salient correlation with the etiology of AD. Gastrointestinal microorganisms prevalent in *Bifidobacteria* and *Lactobacilli* demonstrate the capability for glutamate metabolism, resulting in the synthesis of GABA, a pivotal inhibitory neurotransmitter within the central nervous system. This neurotransmitter assumes a cardinal nexus within the complexities of the central nervous system. The dysregulation encompassing glutamatergic neurotransmission, encapsulating impediments in GABAergic signaling, diminished concentrations of glutamate, and the transcriptional downregulation of pivotal glutamate transporters, remains poised with the potential to act as a harbinger, precipitating cognitive debilitations intrinsic to the realm of AD (Mitew et al., 2013; Paula-Lima et al., 2013).

The hypothalamic–pituitary–adrenal (HPA) axis emerges as a pivotal non-neuronal conduit, facilitating the intricate transmission of information along the microbiota-gut-brain axis. Effectively, the gut microbiota establishes a reciprocal avenue of communication with the brain, mediated by an array of neurotransmitters, including dopamine, GABA, 5-HT, neuropeptides, hormones (such as corticotropin-releasing hormone secreted by the HPA axis), and SCFAs (Sorboni et al., 2022). This orchestration fosters a multifaceted interplay between the cerebral and intestinal microbial domains. The mechanistic influence of gut microbiota, hinging upon the agency of GLP-1 receptors, exerts its dominion over hypothalamic inflammatory processes. This incited inflammation begets an elevated expression profile of inflammatory mediators, neuronal compromise, and reactive gliosis characterized by the enlistment, proliferation, and activation of microglia and astrocytes. This complex interplay potentially acts as a harbinger for the trajectory of AD (Thaler et al., 2012; Heiss et al., 2021; Milligan Armstrong et al., 2021).

Amidst the realm of AD, perturbations and maladjustments within the HPA axis materialize as escalated basal cortisol levels, heightened corticosteroid concentrations, and aberrant modulation of glucocorticoid feedback mechanisms (mediated by glucocorticoid receptors within the hippocampus, along with atypical hypothalamic and anterior pituitary feedback loops). In murine models, there is also evidence of augmented corticosterone levels. Moreover, heightened glucocorticoid concentrations perpetuate persistent activation of glucocorticoid receptors, accompanied by stress-induced compromise of hippocampal neurons, ultimately culminating in sequences of neurodegeneration (Hebda-Bauer et al., 2013; Morgese et al., 2017; Justice, 2018). This elevation of glucocorticoids precipitates the protracted activation of GR, thereby imparting considerable stress-induced damage upon hippocampal neurons, and subsequently paving the path for ensuing neurodegenerative alterations (Zhang et al., 2017). Notably, select cellular factors released by the gut microbiota, exemplified by LPS and peptidoglycans, emerge as potent instigators, skillfully inciting the functional impairment of the HPA axis. This orchestrated impairment bears a notable contribution to the complex landscape of AD pathology. In contrast, probiotics of the *Lactobacillus* and *Bifidobacterium* lineages exhibit the potential to ameliorate HPA axis dysfunctions prompted by stress. This amelioration consequently augments the domains of learning, cognition, and manifestations of psychopathology (Vakharia and Hinson, 2005; Richards et al., 2006; Desbonnet et al., 2010). Furthermore, in concert with their engagement with receptors situated on colonic epithelial cells, SCFAs prompt a consequential retort by provoking enteroendocrine L cells to discharge GLP-1 and peptide YY, alongside sundry other gastrointestinal hormones. This orchestrated sequence expedites the conveyance of indirect cues to the cerebral sphere via the systemic circulatory route or the vagal nerve conduit. Thereafter, these bioactive compounds, operating in a mutually influential fashion, exert a conspicuous influence on cognitive capacities, memory retention, and affective tendencies (Tolhurst et al., 2012; Psichas et al., 2015; Larraufie et al., 2018). Such metabolic byproducts effectively partake in the synthesis of enzymes, modulation of host metabolism, and transmission of bioactive signals. Remarkably, their influence even extends to cognitive function and behavioral manifestations within the brain, facilitated by means such as vagus nerve stimulation or modulation of the enteric nervous system (Fan and Pedersen, 2021).

#### 4.2.5. Blood–brain barrier

The intricate interplay of the gut microbiome extends its regulatory reach to encompass cerebral substrate exchange and the orchestration of inflammatory cascades within the central nervous system. This regulatory prowess finds its nexus in the nuanced modulation of the blood–brain barrier’s permeability. The luminal domain of the gastrointestinal milieu becomes host to a cohort of molecules of intricate nature, among which lipopolysaccharides (LPS), colloquially known as endotoxins, reign preeminent. These moieties, architecturally embellishing the extracellular matrices of bacterial phyla, find their favored abode within the enclave of select Gram-negative taxa that colonize the intestinal fiefdom. Exhibiting an affinity for prodigious LPS production, these bacterial constituents engage in the synthesis of amyloid-like proteins, concomitantly generating signaling moieties germane to LPS/amyloid-associated cascades. Plausibly implicated in the modulation of signal transduction pathways and the instigation of pro-inflammatory cytokine milieu germane to the pathogenic trajectory of AD, these molecular parleys assume a pivotal role. The physiological function of LPS within the gastrointestinal tract primarily assumes the role of an immunomodulatory stimulus, eliciting inflammatory responses aimed at countering the threat posed by exogenous microbial colonies. However, meticulous investigations have unveiled the presence of bacterial LPS within cerebral lysates of the hippocampal and neocortical regions in brains afflicted by AD (Zhao et al., 2017c). This phenomenon is postulated to originate from processes associated with aging, vascular irregularities, or neurodegenerative pathologies, culminating in the plausible “leakage” of neurotoxic constituents into the systemic circulation and the cerebral vascular milieu. This intricate sequence culminates in the accumulation of these entities at both systemic and cerebral strata. This sequence of events may trigger the amplification of reactive oxygen species, concurrently activating the NF-κB signal transduction pathway. Consequently, there is an induction in the upregulation of the pro-inflammatory miRNA-34a, instigating a reduction in TREM2 expression. This reduction subsequently hampers the phagocytic efficacy of microglial cells, thereby fostering the buildup of Aβ aggregates. Furthermore, the presence of bacterial-derived LPS and amyloidogenic proteins serves to exacerbate the permeability of the intestinal barrier. This exacerbation further augments the quantities of cytokines and other smaller pro-inflammatory moieties, including but not limited to IL-17A and IL-22. These entities have been intimately linked with the pathogenesis of AD. Additionally, it is noteworthy that LPS exhibits a specific propensity to engage with TLR4, consequently giving rise to the production of multifaceted cytokines and chemokines. These molecules, in turn, intricately coordinate processes of inflammation, as well as innate and subsequent adaptive immune responses (Rhee, 2014; Zhao and Lukiw, 2015; Zhao et al., 2017a,b).

Dysbiosis within the gut microbiota, characterized by a decline in both the diversity and abundance of microbial species, inflammation, and the presence of toxic substances, has the potential to disrupt the delicate equilibrium of the IB. The repercussions of such disruption are twofold: inflammation either originates at the interface of the IB or gains momentum subsequent to its compromise, ultimately disseminating along its trajectory and significantly contributing to the development of a spectrum of prevalent chronic diseases (Desai et al., 2016; Mou et al., 2022). Unsurprisingly, this state of sustained systemic inflammation engenders structural alterations within the BBB. Preceding the decline in integrity observed within the IB and BBB, certain minuscule molecules, derived from the metabolic activities of pathogenic gut symbionts, traverse their way into the brain. The infiltration of these molecules serves as the impetus for the initiation of central nervous system inflammation, thus perpetuating a vicious cycle of neuroinflammatory responses (Kurita et al., 2020).

## 5. Therapeutic strategies targeting the intestinal microbiota

Given the intricate interrelation between the gut microbiota and AD, delving into the prospects of manipulating the gut microbiota as a therapeutic approach for AD treatment represents a promising trajectory for pioneering insights (Table 1). In contrast to conventional pharmacotherapies that predominantly focus on brain-centric targets, interventions centered on the gut microbiota proffer discernible advantages: (i) Precision: therapeutic modalities targeting the gut microbiota offer a means to exert direct influence over the intricate structure and operational dynamics of intestinal microorganisms, obviating the need to surmount the formidable BBB. By directly intervening in the interplay between the gut microbiota and the host, concerns pertaining to the drug’s capacity to traverse the BBB are nullified, thereby facilitating meticulous modulation of the microbiota-brain axis. (ii) Safety: targeting the cerebral domain through pharmacological interventions presents inherent safety concerns and confronts obstacles pertaining to drug metabolism. In stark contrast, strategies that govern the composition of the intestinal microbiota are widely acknowledged for their comparatively benign nature. By manipulating dietary patterns and employing modalities encompassing probiotics, prebiotics, or analogous agents for gut microbiota modulation, the attainment of microbiota regulation can be realized devoid of appreciable induction of deleterious repercussions. (iii) Versatility: the impact of the gut microbiota extends far beyond neurological disorders, exerting its influence on a multitude of dimensions pertaining to human health. Through targeted modulation of the gut microbiota, notable enhancements can be observed in the regulation of the immune system, human metabolism, and the holistic state of well-being. Therefore, therapeutic interventions targeting the gut microbiota have the potential not only to address AD itself but also to alleviate associated complications such as insulin resistance and abnormalities in lipid metabolism.

**Table 1:** Therapeutic intervention of Alzheimer’s disease through gut microbiota manipulation.

Category	Drug	Effect	References
Traditional Chinese medicine and extracts	Quercetin	Mitigating filamentous bacterial populations; mitigating neuroinflammatory responses induced by Aβ plaques; enhancing BDNF levels.	Lv et al. (2018) and Yang et al. (2020)
	Xanthoceraside	Modulating the *Firmicutes*/*Bacteroidetes* ratio; exerting an influence over the metabolic processes implicated in AD.	Zhou et al. (2019)
	Ginseng	Restoring gut microbiota dysbiosis in AD; eliciting effects conducive to attenuating the aging process; augmenting cognitive capacities via TLR Pathways.	Zhao et al. (2014) and Wang et al. (2020)
	Curcumin	Modulating the relative abundance of the gastrointestinal microbiota; mitigating hippocampal amyloid-beta deposition; enhancing spatial learning and memory capabilities.	Sun Z. Z. et al. (2020)
	Jatrorrhizine	Mitigating gut dysbiosis; alleviating memory deficits.	Wang S. et al. (2019)
	Rhodiola glycoside	Improving intestinal barrier functionality; modulating gut microbiota population; regulating inflammation within the peripheral and CNS.	Xu et al. (2019) and Xie et al. (2020)
	Scutellaria baicalensis	Modulation of lipid and glucose metabolism; altering gut microbiota composition and activity, along with subsequent shifts in their ensuing metabolomic profiles; enhancing cognitive faculties pertaining to AD.	Shi et al. (2023)
	Prepared Rehmannia root	Reversing gut dysbiosis in AD mice; mitigation of cognitive decline.	Meng et al. (2017)
	*Tetragonia tetragonioides* Kuntze	Modulating the gut microbiota; enhancing insulin resistance; attenuating Aβ deposition in AD.	Choi et al. (2016) and Kim et al. (2020)
	Morinda officinalis	Sustaining gut microbiota diversity and stability in AD mice; improving neuronal function, oxidative stress, and inflammatory disruptions associated with cognitive impairment.	Chen et al. (2017) and Xin et al. (2019)
Traditional Chinese medicine formulations	Huanglian-Jiedu decoction	Restoring BA equilibrium; augmenting of SCFAs concentrations; suppressing aberrations in lipid metabolism; modulating NMDAR-mediated glutamatergic transmission alongside pathways implicated in adenosine-associated signaling; reducing of Aβ deposition; enhancing cognitive faculties.	Liu et al. (2019), Fan et al. (2021), and Wang L. et al. (2022)
	Liuwei-Dihuang Decoction	Modulating the gut microbiota composition; improves cognitive impairment.	Wang et al. (2016) and Wang J. et al. (2019)
	Foshou San	Modulation of alkaline phosphatase and the gut microbiota via the intricate gut-liver-brain axis; ameliorating of systemic inflammation and oxidative stress orchestrated by LPS.	Lu et al. (2019)
	Yizhi-Anshen Granules	Balancing microbial richness and diversity within individuals afflicted by MCI; mitigating cognitive decline.	Yue et al. (2019)
	Chaihu-Shugan-San	Modulating intestinal inflammation and the microbiota, the induction of NF-κB-mediated BDNF expression is achieved; improving neuronal damage, synaptic impairment, and Aβ deposition.	Han et al. (2021) and Li et al. (2023a)
Dietary ways	Mediterranean diet	Enhancing microbiota dysbiosis AD and attenuating the risk of AD onset.	Nagpal et al. (2019) and Solch et al. (2022)
	Ketogenic diet	Minimizing *Bifidobacteria* abundance; reducing pro-inflammatory cell levels; improving cerebral vasculature and BBB functionality.	Gasior et al. (2006), Ang et al. (2020), and Xu et al. (2022)
	Fermented food products	Amplifying the relative abundance of microflora; Alleviating inflammatory mediators.	Wastyk et al. (2021)
	Dietary inulin	Elevating gastrointestinal microbiota metabolites, including SCFAs, tryptophan-derived metabolites, and BA; reducing hippocampal inflammation gene expression.	Hoffman et al. (2019)
Probiotic	Probiotic formulation (SLAB51)	Diminishing Aβ aggregation and partially restore compromised neuronal proteins.	Bonfili et al. (2017)
	*Bifidobacterium breve* strain A1	Diminishing the expression of inflammation and immune response genes in the hippocampus.	Kobayashi et al. (2017)
	Probiotic supplementation (BGN4 and BORI)	Heighten the serum levels of BDNF.	Kim et al. (2021)
Wide-spectrum courses of antibiotic treatment	Antibiotic cocktail	Altering the gut microbiome to mitigate Aβ deposition.	Minter et al. (2016) and Dodiya et al. (2020)
Fecal microbiota transplantation	Transplanting healthful gut microbiota	Mitigating burden of Aβ plaques; diminishing levels of soluble Aβ40 and Aβ42; changing cognitive deficits.	Sun et al. (2019)

AD, Alzheimer’s disease; Aβ, amyloid beta; BA, bile acids; BBB, blood–brain barrier; BDNF, blood–brain-derived neurotrophic factor; CNS, central nervous systems; LPS, lipopolysaccharides; MCI, mild cognitive impairment; SCFAs, short-chain fatty acids; TLR, toll-like receptors.

### 5.1. Traditional Chinese medicine and extracts

In recent investigations, the pragmatic application of traditional Chinese medicine (TCM) formulations has unveiled notable advantages in comparison to other pharmaceutical agents. These advantages encompass their intricate compound composition, facilitating the concurrent modulation of multiple targets, while showcasing minimal adverse effects and augmented biocompatibility. By intervening through diverse pathways, TCM interventions demonstrate efficacy in effectively regulating the composition of the gut microbiota, thereby ameliorating the microecological milieu of the intestines. Consequently, this alleviates the pathological conditions associated with central nervous system disorders, ultimately augmenting therapeutic outcomes (Karageorgis et al., 2014; Gu and Lai, 2020; Atanasov et al., 2021; Dai et al., 2022; Li et al., 2023b). A plethora of research findings corroborate the efficacy of specific TCM monomers, extracts, TCM formulae, and TCM combinations in modulating the gut microbiota, including its composition, diversity, and abundance. These interventions have demonstrated a significant preventive and therapeutic impact on AD.

Currently, extensive research has revealed that specific components and combinations of TCM possess the capacity to modulate the gut microbiota, thereby directly or indirectly enhancing AD. Quercetin, a flavonoid abundant in various plant-based foods, primarily enhances gut microbiota diversity, reduces filamentous bacteria, mitigates neuroinflammation induced by Aβ plaques, and upregulates brain-derived neurotrophic factors. Collectively, these mechanisms ameliorate cognitive impairment in AD (Lv et al., 2018; Yang et al., 2020). Fecal microbiota transplantation studies have documented the potent anti-AD activity of Xanthoceraside, a naturally occurring compound extracted from the husk of *Xanthoceras sorbifolia*. Xanthoceraside adjusts the *Firmicutes*/*Bacteroidetes* ratio, influencing metabolic processes implicated in AD and other neurological disorders (Zhou et al., 2019). Ginseng, a renowned herbal remedy for neurodegenerative diseases, contains ginsenoside Rg1 as its primary active constituent (Yu et al., 2007). Apart from rectifying gut microbiota dysbiosis in AD, ginsenoside Rg1 exerts anti-aging and cognitive-enhancing effects through TLR pathways (Zhao et al., 2014; Wang et al., 2020). Curcumin, a polyphenolic compound derived from turmeric, represents a promising natural compound with anti-AD properties. It modulates the relative abundance of gut microbiota, alleviates hippocampal Aβ deposition, and improves spatial learning and memory in AD mice (Sun Z. Z. et al., 2020). Jatrorrhizine, an isoquinoline alkaloid extracted from the Chinese herb Coptis chinensis, addresses gut dysbiosis in AD mice, ameliorating memory deficits. Furthermore, berberine exhibits antimicrobial properties and is commonly employed for detoxification and anti-hyperglycemic purposes (Wang S. et al., 2019). Rhodiola glycoside, a major bioactive component extracted from *Rhodiola rosea*, exerts a preventive effect on cognitive changes in mice by enhancing gut barrier function, modulating gut microbiota abundance, and regulating peripheral and central nervous system inflammation (Xu et al., 2019; Xie et al., 2020). Baicalein, an active compound derived from Scutellaria baicalensis root, potentially influences lipid and glucose metabolism through its impact on gut microbiota and their metabolites, leading to improvements in AD cognition (Shi et al., 2023). Prepared Rehmannia root, derived from the dried roots of Rehmannia glutinosa, undergoes alcohol steaming to enhance its medicinal properties (Su et al., 2023). The primary components of prepared Rehmannia root may synergistically reverse gut dysbiosis in AD mice, thereby ameliorating cognitive impairment (Meng et al., 2017). *Tetragonia tetragonioides* Kuntze, also known as New Zealand spinach, contains phospholipids, a complex of vitamin A and B, and pectin polysaccharides. It improves insulin resistance and reduces Aβ deposition in AD by modulating the gut microbiota (Choi et al., 2016; Kim et al., 2020). Morinda officinalis, a traditional Chinese herb commonly known as “Ba Ji Tian,” encompasses multiple active constituents. Among them, oligosaccharides maintain gut microbiota diversity and stability in AD mice, improving neuronal function, oxidative stress, and inflammatory disruptions associated with cognitive impairment (Chen et al., 2017; Xin et al., 2019). Besides the aforementioned TCM interventions, it is plausible that numerous undisclosed or yet-to-be-discovered natural compounds with even greater therapeutic efficacy and minimal side effects exist.

### 5.2. Traditional Chinese medicine formulations

In addition to isolated constituents and chemical entities, TCM formulations exemplify remarkable regulatory impacts on the gut microbiota while concurrently targeting precise complexities. Illustratively, the Huanglian-Jiedu Decoction, administered within a context of AD murine models induced by a high-fat regimen, not only effects the restoration of BA equilibrium and augmentation of SCFAs concentrations, but also exerts suppression upon aberrations in lipid metabolism and counteracts inflammation stemming from the high-fat dietary milieu (Fan et al., 2021; Wang L. et al., 2022). Moreover, it orchestrates modulation of NMDAR-mediated glutamatergic transmission alongside pathways implicated in adenosine-associated signaling. As a result, attenuation of Aβdeposition is achieved, concomitant with observable enhancements in murine cognitive faculties (Liu et al., 2019). The active compound group of Liuwei-Dihuang Decoction, LW-AFC, or its extracted oligosaccharide component (CA-30), improves cognitive impairment in AD mice by modulating the gut microbiota composition (Wang et al., 2016; Wang J. et al., 2019). The time-honored TCM formulation, Foshou San, which has been employed in China for countless centuries, exhibits remarkable efficacy in the modulation of alkaline phosphatase and the gut microbiota via the intricate gut-liver-brain axis. Through its discernible amelioration of systemic inflammation and oxidative stress orchestrated by LPS, Foshou San substantiates a discerning mitigation of the concomitant pathology observed within a murine model of AD (Lu et al., 2019). The effects of Yizhi-Anshen Granules, a TCM formulation renowned for its efficacy in mitigating cognitive decline and sleep disturbances, are plausibly mediated through the orchestration of microbial richness and diversity within individuals afflicted by MCI (Yue et al., 2019). Chaihu-Shugan San (CSS), a well-known traditional herbal formula with liver-soothing and depression-relieving properties, demonstrates potential in preventing and treating AD. CSS is believed to modulate intestinal inflammation, gut microbiota composition, induce NF-κB-mediated BDNF expression, and improve neuronal damage, synaptic impairment, and Aβ deposition in the mouse brain, contributing to its therapeutic mechanisms (Han et al., 2021; Li et al., 2023a).

### 5.3. Dietary influence

The pivotal determinant in the orchestration of gut microbiota assembly and genetic constitution resides within the dietary milieu. Distinctive edibles or dietary paradigms harbor the potential to exert a discernible impact upon the assortment and prevalence of a myriad of bacterial taxa inhabiting the gastrointestinal milieu. In doing so, they concurrently uphold the equilibrium of the host’s internal milieu.

The adherence to the Mediterranean dietary regimen, characterized by its robust incorporation of legumes, cereals, fruits, and vegetables, moderated consumption of fish and dairy, and controlled intake of meat products, demonstrates a notable capacity for modulating the aggregation of Aβ (Roman et al., 2019; Solch et al., 2022). Simultaneously, it exerts regulatory effects on the progression of AD through the augmentation of commensal gut microflora. Through meticulous investigation, it has come to light that maintaining a strict adherence to the tenets of the Mediterranean dietary paradigm yields a remarkable 41% reduction in the susceptibility to the onset of AD (Solch et al., 2022). This intriguing phenomenon can potentially be attributed to the diet’s inherent propensity for nurturing bacterial strains that typically experience diminishment in the context of AD. Exemplars of such strains include *Ruminococcus*, *Akkermansia muciniphila*, and selective cohorts of butyrate-producing bacteria (Nagpal et al., 2019). Recent inquiries have unveiled that the ketogenic dietary paradigm (marked by significantly reduced carbohydrate consumption and heightened lipid utilization) harbors the potential to alleviate symptomatic presentations across an array of neurodegenerative conditions, spanning ailments like AD and Parkinson’s disease (Gasior et al., 2006). Ang et al. (2020), in their observations, have discerned that the ketogenic dietary framework preferentially diminishes the population of bifidobacteria within the intestinal microbiota and curtails the quantities of pro-inflammatory Th17 cells. Meanwhile, Xu et al. (2022), having subjected AD-affected murine models to a four-month regimen of ketogenic dietary intervention, have unearthed that the ketogenic diet induces mitigation of cerebral cognitive impairment by attenuating the deposition of amyloid-beta, activation of glial cells, and neuroinflammatory responses. Additionally, the study conducted by Wastyk et al. (2021), entailing a 10-week regimen of high-fermentation/high-fiber nourishment, has observed that a diet rich in fiber amplifies the relative abundance of microflora while dampening inflammatory markers, encompassing IL-6, IL-10, and other pro-inflammatory cytokines, during the course of heightened-fermentation dietary intervention. Such modifications potentially ameliorate the diminished abundance of intestinal microbes and the surplus of inflammatory mediators emblematic of AD, thereby mitigating cognitive impairment. Beyond the preceding dietary framework, there are specific alimentary constituents and gastronomic traditions evincing prophylactic efficaciousness against AD (Hoffman et al., 2019). While the body of research underscores the diverse effects of disparate dietary paradigms on cerebral afflictions, it remains imperative for further investigations to expound the precise mechanisms through which diets and their constituents exert influence on the microbiota-gut-brain axis. This exploration is vital to ascertain whether dietary interventions targeting the microbiota genuinely incite transformative changes in overall cerebral functionality.

### 5.4. Probiotic

Probiotic agents, residing microorganisms bestowing health advantages upon their host, have garnered mounting attention due to their aptitude for modulating cerebral wellness through the manipulation of the gut microbiota milieu. A convergence of analytical scrutiny underscores the potential of probiotics to enhance cognitive aptitudes among individuals contending with AD or MCI, a feat accomplished by mitigating inflammatory and oxidative biomarkers (Den et al., 2020). Significant within this context resides the inquiry conducted by Bonfili et al., wherein the utilization of the SLB51 probiotic formulation exhibited the capacity to choreograph the composition of the gut microbiome and concentrations of plasma metabolites (Bonfili et al., 2017). This orchestration, in turn, led to the mitigation of Aβ aggregate accumulation. This phenomenon, concomitant with the partial restoration of the neuronal protein autophagy pathway, garnered particular prominence within the milieu of early-stage murine models of AD. In a similar vein, the investigations by Kobayashi et al. illuminated the remedial characteristics inherent in the *Bifidobacterium breve A1 strain*, a distinct probiotic variant specialized in assuaging hippocampal inflammation in AD-affected mice (Kobayashi et al., 2017). Moreover, this intervention elicited a noticeable attenuation in the expression of genes governing immune responses. Parallel to these discernments, the undertaking by Kim et al. encompassed the dispensation of BGN4 and BORI probiotics to a geriatric cohort, thereby unveiling a marked elevation in the serum concentrations of cerebral-origin neurotrophic factors (Kim et al., 2021). This augmentation in neurotrophic factors fostered a safeguarding milieu that bolstered and nurtured neuronal viability, consequently retarding the pathological advancement emblematic of AD. Notwithstanding, it is manifest that a call for heightened precision, sensitivity, and dependability in detection methodologies is palpable, mandating a surge in experimental investigations to definitively authenticate the therapeutic potential that underlies probiotics in the domain of interventions for AD.

### 5.5. Other therapeutic approaches

In concert with the aforementioned therapeutic modalities, given the evident correlation linking the consortium of microbiota to the pathogenesis of AD, scholarly endeavors have diligently expanded towards harnessing the manipulation of gastrointestinal microbial milieu as an ancillary strategy for intervening in AD. These interventions encompass extended, wide-spectrum courses of antibiotic treatment, fecal microbiota transplantation (FMT), prebiotic agents, physical exertion, and active involvement in sports activities. The overarching objective of these interventions resides in the amelioration of cognitive functionality coupled with the attenuation of the progression of the ailment.

Significantly, the enduring perturbations observed in the composition and diversity of the intestinal microbial landscape following sustained administration of broad-spectrum antibiotic regimens have demonstrably exhibited an inherent capacity to mitigate the accrual of Aβ. Meticulous observations by investigators have brought to light the transformative impact of a concoction of antibiotic cocktail-mediated perturbations of the gut microbiome in male murine models, precipitating a discernible reduction in Aβ deposition across two discrete transgenic strains (APP_SWE_/PS1_dE9_ and APPPS1-21) (Minter et al., 2016; Dodiya et al., 2020). This underscores, in no uncertain terms, the pivotal role played by dysbiosis in the gut microbiota milieu in the orchestration of Aβ genesis and subsequent sedimentation. These findings accentuate the latent promise harbored within the judicious maintenance of intestinal microbiota equilibrium and the discerning deployment of antibiotic interventions as plausible avenues for heightening the prospects in the realm of AD enhancement.

Fecal microbiota transplantation (FMT), an increasingly prevalent technique for modifying microbial composition, presents an avenue of profound interest. This therapeutic modality entails the transfer of gut microbiota from a donor of “healthful” constitution to an individual harboring a deranged gut microbiota, with the principal objective of rectifying the recipient’s ecological disequilibrium (Matheson and Holsinger, 2023). This strategy has garnered substantial traction as a captivating therapeutic avenue within the domain of neurological ailments. In their investigation, Sun et al. (2019) meticulously probed the ramifications of FMT on murine models possessing the APP/PS1 genetic configuration. In comparison to their untreated counterparts, murine recipients of FMT sourced from wild-type (WT) contributors showcased marked enhancements in cognitive function. These improvements were concomitant with a mitigation in the burden of Aβ plaques, as well as diminished levels of soluble Aβ40 and Aβ42. Notably, a concurrent elevation in the expression of proteins linked to synaptic plasticity was observed, paralleled by a noteworthy amplification in the presence of propitious SCFAs, most notably butyrate, within the intestinal milieu (Sun et al., 2019). In analogous vein, Fujii et al. reported analogous findings; WT mice subjected to FMT from human donors afflicted with AD, particularly at an earlier age, displayed discernible cognitive deficits relative to those subjected to FMT from donors evincing sound health (Fujii et al., 2019).

## 6. Conclusions and future perspectives

After a protracted span of assiduous inquiry spanning numerous decades, the intricate interplay linking the gut microbiota with the formidable malaise of AD has borne forth a plenitude of noteworthy headway. The arc of research, having transitioned from its nascent stages of clinical observations, has progressively unfurled to unveil a more intricate and nuanced expedition into the substratal mechanisms that underlie this affliction. Presently, this scholarly odyssey finds itself embarked upon an inexorable trajectory, wherein the elucidation of causal nexuses stands as an imperious goal. The present subject expounds comprehensively upon the intimate intertwinement existing between the dysbiosis inherent to the gut microbiota and the distinctive physiological perturbations that hallmark AD. The gut microbiota, much like a central protagonist bestowed with significant eminence, assumes a twofold role—both direct and indirect—exerting its sway across the evolving narrative of AD distressing trajectory. Beyond this role, it bestows upon us the tantalizing potentiality of harnessing its agency as a propitious vector in the pursuit of prospective therapeutic interventions for AD. Notwithstanding these revelations, the labyrinthine etiological landscape characterizing AD, by its very nature, compels the relentless quest for more robust and discerning biological markers, as well as the development of pragmatic modalities for treatment, both of which ascend to a zenith of paramount import.

A profound grasp of the underlying pathogenic substratum inherent to AD serves to not only enhance our ability to navigate intricate therapeutic challenges but also to usher in unprecedented perspectives for comprehending the multifaceted interplay between the gut microbiota and AD pathology. While a definitive explication elucidating the precise mechanistic underpinnings of the symbiotic axis that connects the gut microbiota with the cerebral aggregation of pathological Aβproteins still evades us, a spectrum of hypotheses and nascent investigative forays present enlightening trajectories. Within this spectrum, considerations encompass inflammatory cascades, perturbations in metabolism, immunomodulatory interchanges, neurotransmitter transmission, and the dynamic contributions of the BBB. Nonetheless, the trajectory toward more decisive substantiation necessitates a profound expedition into myriad enigmatic factors and labyrinthine biological pathways. Chief among these is the imperious imperative to delve further into discerning the plausible direct correlations between particular microbial strains or consortia and the cerebral accrual of Aβ, alongside the explication of their impact upon Aβ metabolism via intermediary metabolites, immune modulatory influences, or alternative mechanisms. Moreover, the unveiling of signaling cascades within the gut-brain axis, encompassing neuroactive compounds, peptides, and metabolites, portends an amplification of our sagacity regarding the sway wielded by the gut microbiota upon cerebral homeostasis and the trajectory of morbid progression. Yet, circumspection remains warranted, given that this specific realm of inquiry abides within its nascence, replete with extant constraints. Moreover, while instances of solitary-case exploration might germinate prospective inquiry, animal model experiments conduce to the profundity of our apprehension; notwithstanding, formidable encumbrances endure within the sphere of clinical trials, intermittently materializing as absolute dearth.

Moreover, delineating a definitive profile of a healthy microflora at this juncture poses significant challenges, owing to interindividual variations in both the abundance and species diversity of gut microbiota. Future investigations ought to delve into the structural patterns and strain-level regularities of gut microbiota in individuals afflicted with AD, employing metagenomic analysis and integrating multiple omics approaches, including proteomics, genomics, and metabolomics. This approach surpasses the sole reliance on 16S rRNA gene sequencing. In the context of microbial intervention for high-risk populations susceptible to AD, such as children, immunocompromised patients, and the elderly, it becomes imperative to incorporate additional studies that probe into the effects of microbial therapeutic interventions. Attention should be given to potential interactions with concurrent treatments, appropriate sample sizes, and extended follow-up studies. Furthermore, it is crucial to consider the impact of medications on other microbial interventions during administration.

It is noteworthy that while therapeutic approaches targeting the gut microbiota offer certain advantages, the current strategies for modulating the gut microbiota in AD are still in the realm of research. Although preliminary research findings suggest the potential benefits of gut microbiota modulation in the context of AD, a more comprehensive array of studies is indispensable to ascertain the most efficacious intervention methods and their long-term effects. Such endeavors will pave the way for the meticulous design of interventions based on gut microbiota modulation or the utilization of specific active components, thereby facilitating a deeper comprehension of the underlying mechanisms and the development of effective and safe approaches for both the prevention and treatment of AD.

## Author contributions

JL: writing – original draft. JL, XD, and YW: data collection and integration. BL, WC, NZ, and HZ: conceptualization. BL, XD, and NZ: supervision. BL and NZ: project administration. WC and HZ: funding acquisition. All authors contributed to the article and approved the submitted version.

## Funding

This work was financially supported by Natural Science Foundation of Heilongjiang Province of China (no. LH2023H073).

## Conflict of interest

The authors declare that the research was conducted in the absence of any commercial or financial relationships that could be construed as a potential conflict of interest.

## Publisher’s note

All claims expressed in this article are solely those of the authors and do not necessarily represent those of their affiliated organizations, or those of the publisher, the editors and the reviewers. Any product that may be evaluated in this article, or claim that may be made by its manufacturer, is not guaranteed or endorsed by the publisher.

## References

[ref1] Acosta C. Anderson H. D. Anderson C. M. (2017). Astrocyte dysfunction in Alzheimer disease. J. Neurosci. Res. 95, 2430–2447. doi: 10.1002/jnr.24075, PMID: 28467650

[ref2] Adak A. Khan M. R. (2019). An insight into gut microbiota and its functionalities. Cell. Mol. Life Sci. 76, 473–493. doi: 10.1007/s00018-018-2943-4, PMID: 30317530 PMC11105460

[ref3] Agirman G. Hsiao E. Y. (2021). SnapShot: the microbiota-gut-brain axis. Cells 184:e2521. doi: 10.1016/j.cell.2021.03.022 33930299

[ref4] Agus A. Planchais J. Sokol H. (2018). Gut microbiota regulation of tryptophan metabolism in health and disease. Cell Host Microbe 23, 716–724. doi: 10.1016/j.chom.2018.05.003, PMID: 29902437

[ref5] Akhtar A. Sah S. P. (2020). Insulin signaling pathway and related molecules: role in neurodegeneration and Alzheimer's disease. Neurochem. Int. 135:104707. doi: 10.1016/j.neuint.2020.104707, PMID: 32092326

[ref6] Alexeev E. E. Lanis J. M. Kao D. J. Campbell E. L. Kelly C. J. Battista K. D.. (2018). Microbiota-derived indole metabolites promote human and murine intestinal homeostasis through regulation of Interleukin-10 receptor. Am. J. Pathol. 188, 1183–1194. doi: 10.1016/j.ajpath.2018.01.011, PMID: 29454749 PMC5906738

[ref8] Alzheimer’s disease facts and figures (2023). Alzheimer’s disease facts and figures. Alzheimers Dement. 19, 1598–1695. doi: 10.1002/alz.13016 36918389

[ref7] Ang Q. Y. Alexander M. Newman J. C. Tian Y. Cai J. Upadhyay V.. (2020). Ketogenic diets Alter the gut microbiome resulting in decreased intestinal Th17 cells. Cells 181:e1216. doi: 10.1016/j.cell.2020.04.027 PMC7293577 32437658

[ref9] Arumugam M. Raes J. Pelletier E. Le Paslier D. Yamada T. Mende D. R.. (2011). Enterotypes of the human gut microbiome. Nature 473, 174–180. doi: 10.1038/nature09944, PMID: 21508958 PMC3728647

[ref10] Atanasov A. G. Zotchev S. B. Dirsch V. M. The International Natural Product Sciences Taskforce Supuran C. T. (2021). Natural products in drug discovery: advances and opportunities. Nat. Rev. Drug Discov. 20, 200–216. doi: 10.1038/s41573-020-00114-z, PMID: 33510482 PMC7841765

[ref11] Backhed F. Ley R. E. Sonnenburg J. L. Peterson D. A. Gordon J. I. (2005). Host-bacterial mutualism in the human intestine. Science 307, 1915–1920. doi: 10.1126/science.1104816, PMID: 15790844

[ref12] Baloni P. Funk C. C. Yan J. Yurkovich J. T. Kueider-Paisley A. Nho K.. (2020). Metabolic network analysis reveals altered bile acid synthesis and metabolism in Alzheimer's disease. Cell Rep Med 1:100138. doi: 10.1016/j.xcrm.2020.100138, PMID: 33294859 PMC7691449

[ref13] Barka E. A. Vatsa P. Sanchez L. Gaveau-Vaillant N. Jacquard C. Meier-Kolthoff J. P.. (2016). Taxonomy, physiology, and natural products of Actinobacteria. Microbiol. Mol. Biol. Rev. 80, 1–43. doi: 10.1128/MMBR.00019-15, PMID: 26609051 PMC4711186

[ref14] Blair L. J. Frauen H. D. Zhang B. Nordhues B. A. Bijan S. Lin Y. C.. (2015). Tau depletion prevents progressive blood-brain barrier damage in a mouse model of tauopathy. Acta Neuropathol. Commun. 3:8. doi: 10.1186/s40478-015-0186-2 25775028 PMC4353464

[ref15] Blazquez E. Velazquez E. Hurtado-Carneiro V. Ruiz-Albusac J. M. (2014). Insulin in the brain: its pathophysiological implications for states related with central insulin resistance, type 2 diabetes and Alzheimer's disease. Front Endocrinol (Lausanne) 5:161. doi: 10.3389/fendo.2014.00161, PMID: 25346723 PMC4191295

[ref16] Bonaz B. Sinniger V. Pellissier S. (2017). The Vagus nerve in the neuro-immune Axis: implications in the pathology of the gastrointestinal tract. Front. Immunol. 8:1452. doi: 10.3389/fimmu.2017.01452 29163522 PMC5673632

[ref17] Bonfili L. Cecarini V. Berardi S. Scarpona S. Suchodolski J. S. Nasuti C.. (2017). Microbiota modulation counteracts Alzheimer's disease progression influencing neuronal proteolysis and gut hormones plasma levels. Sci. Rep. 7:2426. doi: 10.1038/s41598-017-02587-2 28546539 PMC5445077

[ref18] Braniste V. Al-Asmakh M. Kowal C. Anuar F. Abbaspour A. Toth M.. (2014). The gut microbiota influences blood-brain barrier permeability in mice. Sci. Transl. Med. 6:263ra158. doi: 10.1126/scitranslmed.3009759, PMID: 25411471 PMC4396848

[ref19] Brunt V. E. Larocca T. J. Bazzoni A. E. Sapinsley Z. J. Miyamoto-Ditmon J. Gioscia-Ryan R. A.. (2021). The gut microbiome-derived metabolite trimethylamine N-oxide modulates neuroinflammation and cognitive function with aging. Geroscience 43, 377–394. doi: 10.1007/s11357-020-00257-2, PMID: 32862276 PMC8050157

[ref20] Cai Z. Qiao P. F. Wan C. Q. Cai M. Zhou N. K. Li Q. (2018). Role of blood-brain barrier in Alzheimer's disease. J. Alzheimers Dis. 63, 1223–1234. doi: 10.3233/JAD-180098, PMID: 29782323

[ref21] Cani P. D. Neyrinck A. M. Fava F. Knauf C. Burcelin R. G. Tuohy K. M.. (2007). Selective increases of bifidobacteria in gut microflora improve high-fat-diet-induced diabetes in mice through a mechanism associated with endotoxaemia. Diabetologia 50, 2374–2383. doi: 10.1007/s00125-007-0791-0, PMID: 17823788

[ref22] Capuron L. Miller A. H. (2011). Immune system to brain signaling: neuropsychopharmacological implications. Pharmacol. Ther. 130, 226–238. doi: 10.1016/j.pharmthera.2011.01.014, PMID: 21334376 PMC3072299

[ref23] Carabotti M. Scirocco A. Maselli M. A. Severi C. (2015). The gut-brain axis: interactions between enteric microbiota, central and enteric nervous systems. Ann. Gastroenterol. 28, 203–209. PMID: 25830558 PMC4367209

[ref24] Casterline B. W. Hecht A. L. Choi V. M. Bubeck Wardenburg J. (2017). The *Bacteroides fragilis* pathogenicity island links virulence and strain competition. Gut Microbes 8, 374–383. doi: 10.1080/19490976.2017.1290758, PMID: 28632016 PMC5570422

[ref25] Cattaneo A. Cattane N. Galluzzi S. Provasi S. Lopizzo N. Festari C.. (2017). Association of brain amyloidosis with pro-inflammatory gut bacterial taxa and peripheral inflammation markers in cognitively impaired elderly. Neurobiol. Aging 49, 60–68. doi: 10.1016/j.neurobiolaging.2016.08.019, PMID: 27776263

[ref26] Chang P. V. Hao L. Offermanns S. Medzhitov R. (2014). The microbial metabolite butyrate regulates intestinal macrophage function via histone deacetylase inhibition. Proc. Natl. Acad. Sci. U. S. A. 111, 2247–2252. doi: 10.1073/pnas.1322269111 24390544 PMC3926023

[ref27] Chaudhri O. Small C. Bloom S. (2006). Gastrointestinal hormones regulating appetite. Philos. Trans. R. Soc. Lond. Ser. B Biol. Sci. 361, 1187–1209. doi: 10.1098/rstb.2006.1856 16815798 10.1098/rstb.2006.1856 PMC1642697

[ref28] Chen D. Yang X. Yang J. Lai G. Yong T. Tang X.. (2017). Prebiotic effect of Fructooligosaccharides from Morinda officinalis on Alzheimer's disease in rodent models by targeting the microbiota-gut-brain Axis. Front. Aging Neurosci. 9:403. doi: 10.3389/fnagi.2017.00403 29276488 PMC5727096

[ref29] Choi H. S. Cho J. Y. Jin M. R. Lee Y. G. Kim S. J. Ham K. S.. (2016). Phenolics, acyl galactopyranosyl glycerol, and lignan amides from *Tetragonia tetragonioides* (pall.) Kuntze. Food Sci. Biotechnol. 25, 1275–1281. doi: 10.1007/s10068-016-0201-9, PMID: 30263405 PMC6049267

[ref30] Chong C. Y. L. Bloomfield F. H. O'sullivan J. M. (2018). Factors affecting gastrointestinal microbiome development in neonates. Nutrients 10:274. doi: 10.3390/nu10030274, PMID: 29495552 PMC5872692

[ref31] Corrêa-Oliveira R. Fachi J. L. Vieira A. Sato F. T. Vinolo M. A. R. (2016). Regulation of immune cell function by short-chain fatty acids. Clin. Transl. Immunol. 5:e73. doi: 10.1038/cti.2016.17, PMID: 27195116 PMC4855267

[ref32] Dai R. Sun Y. Su R. Gao H. (2022). Anti-Alzheimer's disease potential of traditional chinese medicinal herbs as inhibitors of BACE1 and AChE enzymes. Biomed. Pharmacother. 154:113576. doi: 10.1016/j.biopha.2022.113576, PMID: 36007279

[ref33] De Strooper B. Karran E. (2016). The cellular phase of Alzheimer's disease. Cells 164, 603–615. doi: 10.1016/j.cell.2015.12.056, PMID: 26871627

[ref34] Den H. Dong X. Chen M. Zou Z. (2020). Efficacy of probiotics on cognition, and biomarkers of inflammation and oxidative stress in adults with Alzheimer's disease or mild cognitive impairment - a meta-analysis of randomized controlled trials. Aging (Albany NY) 12, 4010–4039. doi: 10.18632/aging.102810, PMID: 32062613 PMC7066922

[ref35] Desai M. S. Seekatz A. M. Koropatkin N. M. Kamada N. Hickey C. A. Wolter M.. (2016). A dietary Fiber-deprived gut microbiota degrades the colonic mucus barrier and enhances pathogen susceptibility. Cells 167, 1339–1353.e1321. doi: 10.1016/j.cell.2016.10.043, PMID: 27863247 PMC5131798

[ref36] Desbonnet L. Garrett L. Clarke G. Kiely B. Cryan J. F. Dinan T. G. (2010). Effects of the probiotic *Bifidobacterium infantis* in the maternal separation model of depression. Neuroscience 170, 1179–1188. doi: 10.1016/j.neuroscience.2010.08.005, PMID: 20696216

[ref37] Ding M. Lang Y. Shu H. Shao J. Cui L. (2021). Microbiota-gut-brain Axis and epilepsy: a review on mechanisms and potential therapeutics. Front. Immunol. 12:742449. doi: 10.3389/fimmu.2021.742449, PMID: 34707612 PMC8542678

[ref38] Dodiya H. B. Frith M. Sidebottom A. Cao Y. Koval J. Chang E.. (2020). Synergistic depletion of gut microbial consortia, but not individual antibiotics, reduces amyloidosis in APPPS1-21 Alzheimer's transgenic mice. Sci. Rep. 10:8183. doi: 10.1038/s41598-020-64797-5 32424118 PMC7235236

[ref39] Dodiya H. B. Kuntz T. Shaik S. M. Baufeld C. Leibowitz J. Zhang X.. (2019). Sex-specific effects of microbiome perturbations on cerebral Aβ amyloidosis and microglia phenotypes. J. Exp. Med. 216, 1542–1560. doi: 10.1084/jem.20182386, PMID: 31097468 PMC6605759

[ref40] D'onofrio G. Sancarlo D. Panza F. Copetti M. Cascavilla L. Paris F.. (2012). Neuropsychiatric symptoms and functional status in Alzheimer's disease and vascular dementia patients. Curr. Alzheimer Res. 9, 759–771. doi: 10.2174/156720512801322582, PMID: 22715983

[ref41] Dupuis J. P. Nicole O. Groc L. (2023). NMDA receptor functions in health and disease: old actor, new dimensions. Neuron 111, 2312–2328. doi: 10.1016/j.neuron.2023.05.002, PMID: 37236178

[ref42] During M. J. Cao L. Zuzga D. S. Francis J. S. Fitzsimons H. L. Jiao X.. (2003). Glucagon-like peptide-1 receptor is involved in learning and neuroprotection. Nat. Med. 9, 1173–1179. doi: 10.1038/nm919, PMID: 12925848

[ref43] Eckburg P. B. Bik E. M. Bernstein C. N. Purdom E. Dethlefsen L. Sargent M.. (2005). Diversity of the human intestinal microbial flora. Science 308, 1635–1638. doi: 10.1126/science.1110591, PMID: 15831718 PMC1395357

[ref44] Elinav E. Strowig T. Kau A. L. Henao-Mejia J. Thaiss C. A. Booth C. J.. (2011). NLRP6 inflammasome regulates colonic microbial ecology and risk for colitis. Cells 145, 745–757. doi: 10.1016/j.cell.2011.04.022, PMID: 21565393 PMC3140910

[ref45] Erny D. Prinz M. (2020). How microbiota shape microglial phenotypes and epigenetics. Glia 68, 1655–1672. doi: 10.1002/glia.23822, PMID: 32181523

[ref46] Falagas M. E. Siakavellas E. (2000). Bacteroides, Prevotella, and Porphyromonas species: a review of antibiotic resistance and therapeutic options. Int. J. Antimicrob. Agents 15, 1–9. doi: 10.1016/S0924-8579(99)00164-8, PMID: 10856670

[ref47] Fan X. Liu B. Zhou J. Gu X. Zhou Y. Yang Y.. (2021). High-fat diet alleviates Neuroinflammation and metabolic disorders of APP/PS1 mice and the intervention with Chinese medicine. Front. Aging Neurosci. 13:658376. doi: 10.3389/fnagi.2021.658376, PMID: 34168550 PMC8217439

[ref48] Fan Y. Pedersen O. (2021). Gut microbiota in human metabolic health and disease. Nat. Rev. Microbiol. 19, 55–71. doi: 10.1038/s41579-020-0433-9, PMID: 32887946

[ref49] Fischer A. Sananbenesi F. Wang X. Dobbin M. Tsai L. H. (2007). Recovery of learning and memory is associated with chromatin remodelling. Nature 447, 178–182. doi: 10.1038/nature05772, PMID: 17468743

[ref50] Fujii Y. Nguyen T. T. T. Fujimura Y. Kameya N. Nakamura S. Arakawa K.. (2019). Fecal metabolite of a gnotobiotic mouse transplanted with gut microbiota from a patient with Alzheimer's disease. Biosci. Biotechnol. Biochem. 83, 2144–2152. doi: 10.1080/09168451.2019.1644149 31327302

[ref51] Furusawa Y. Obata Y. Fukuda S. Endo T. A. Nakato G. Takahashi D.. (2013). Commensal microbe-derived butyrate induces the differentiation of colonic regulatory T cells. Nature 504, 446–450. doi: 10.1038/nature12721, PMID: 24226770

[ref52] Gasior M. Rogawski M. A. Hartman A. L. (2006). Neuroprotective and disease-modifying effects of the ketogenic diet. Behav. Pharmacol. 17, 431–439. doi: 10.1097/00008877-200609000-00009 16940764 PMC2367001

[ref53] Ghasemi R. Haeri A. Dargahi L. Mohamed Z. Ahmadiani A. (2013). Insulin in the brain: sources, localization and functions. Mol. Neurobiol. 47, 145–171. doi: 10.1007/s12035-012-8339-9, PMID: 22956272

[ref54] Giil L. M. Midttun O. Refsum H. Ulvik A. Advani R. Smith A. D.. (2017). Kynurenine pathway metabolites in Alzheimer's disease. J. Alzheimers Dis. 60, 495–504. doi: 10.3233/JAD-170485, PMID: 28869479

[ref55] Grieco M. Giorgi A. Gentile M. C. D'erme M. Morano S. Maras B.. (2019). Glucagon-like Peptide-1: a focus on neurodegenerative diseases. Front. Neurosci. 13:1112. doi: 10.3389/fnins.2019.01112 31680842 PMC6813233

[ref56] Griffin J. W. Bradshaw P. C. (2017). Amino acid catabolism in Alzheimer's disease brain: friend or foe? Oxidative Med. Cell. Longev. 2017:5472792. doi: 10.1155/2017/5472792, PMID: 28261376 PMC5316456

[ref57] Gu S. Lai L. H. (2020). Associating 197 Chinese herbal medicine with drug targets and diseases using the similarity ensemble approach. Acta Pharmacol. Sin. 41, 432–438. doi: 10.1038/s41401-019-0306-9, PMID: 31530902 PMC7470807

[ref58] Guarner F. Malagelada J. R. (2003). Gut flora in health and disease. Lancet 361, 512–519. doi: 10.1016/S0140-6736(03)12489-0, PMID: 12583961

[ref59] Guo T. Zhang D. Zeng Y. Huang T. Y. Xu H. Zhao Y. (2020). Molecular and cellular mechanisms underlying the pathogenesis of Alzheimer's disease. Mol. Neurodegener. 15:40. doi: 10.1186/s13024-020-00391-7 32677986 PMC7364557

[ref60] Han S. K. Kim J. K. Park H. S. Shin Y. J. Kim D. H. (2021). Chaihu-Shugan-san (Shihosogansan) alleviates restraint stress-generated anxiety and depression in mice by regulating NF-kappaB-mediated BDNF expression through the modulation of gut microbiota. Chin. Med. 16:77. doi: 10.1186/s13020-021-00492-5 34391441 PMC8364688

[ref61] Han Y. Wang B. Gao H. He C. Hua R. Liang C.. (2022). Vagus nerve and underlying impact on the gut microbiota-brain Axis in behavior and neurodegenerative diseases. J. Inflamm. Res. 15, 6213–6230. doi: 10.2147/JIR.S384949 36386584 PMC9656367

[ref62] Hardy J. Selkoe D. J. (2002). The amyloid hypothesis of Alzheimer's disease: progress and problems on the road to therapeutics. Science 297, 353–356. doi: 10.1126/science.1072994, PMID: 12130773

[ref63] Hartai Z. Juhász A. Rimanóczy Á. Janáky T. Donkó T. Dux L.. (2007). Decreased serum and red blood cell kynurenic acid levels in Alzheimer's disease. Neurochem. Int. 50, 308–313. doi: 10.1016/j.neuint.2006.08.012, PMID: 17023091

[ref64] Hayashi H. Sakamoto M. Benno Y. (2002). Phylogenetic analysis of the human gut microbiota using 16S rDNA clone libraries and strictly anaerobic culture-based methods. Microbiol. Immunol. 46, 535–548. doi: 10.1111/j.1348-0421.2002.tb02731.x, PMID: 12363017

[ref65] Hebda-Bauer E. K. Simmons T. A. Sugg A. Ural E. Stewart J. A. Beals J. L.. (2013). 3xTg-AD mice exhibit an activated central stress axis during early-stage pathology. J. Alzheimers Dis. 33, 407–422. doi: 10.3233/JAD-2012-121438, PMID: 22976078 PMC3525735

[ref66] Heiss C. N. Manneras-Holm L. Lee Y. S. Serrano-Lobo J. Hakansson Gladh A. Seeley R. J.. (2021). The gut microbiota regulates hypothalamic inflammation and leptin sensitivity in Western diet-fed mice via a GLP-1R-dependent mechanism. Cell Rep. 35:109163. doi: 10.1016/j.celrep.2021.109163, PMID: 34038733

[ref67] Hoffman J. D. Yanckello L. M. Chlipala G. Hammond T. C. Mcculloch S. D. Parikh I.. (2019). Dietary inulin alters the gut microbiome, enhances systemic metabolism and reduces neuroinflammation in an APOE4 mouse model. PLoS One 14:e0221828. doi: 10.1371/journal.pone.0221828, PMID: 31461505 PMC6713395

[ref68] Hold G. L. Pryde S. E. Russell V. J. Furrie E. Flint H. J. (2002). Assessment of microbial diversity in human colonic samples by 16S rDNA sequence analysis. FEMS Microbiol. Ecol. 39, 33–39. doi: 10.1111/j.1574-6941.2002.tb00904.x, PMID: 19709182

[ref69] Honarpisheh P. Reynolds C. R. Blasco Conesa M. P. Moruno Manchon J. F. Putluri N. Bhattacharjee M. B.. (2020). Dysregulated gut homeostasis observed prior to the accumulation of the brain amyloid-beta in Tg2576 mice. Int. J. Mol. Sci. 21:1711. doi: 10.3390/ijms21051711, PMID: 32138161 PMC7084806

[ref70] Hosoi T. Okuma Y. Matsuda T. Nomura Y. (2005). Novel pathway for LPS-induced afferent vagus nerve activation: possible role of nodose ganglion. Auton. Neurosci. 120, 104–107. doi: 10.1016/j.autneu.2004.11.012, PMID: 15919243

[ref71] Hoyles L. Snelling T. Umlai U. K. Nicholson J. K. Carding S. R. Glen R. C.. (2018). Microbiome-host systems interactions: protective effects of propionate upon the blood-brain barrier. Microbiome 6:55. doi: 10.1186/s40168-018-0439-y 29562936 PMC5863458

[ref72] Hugon P. Dufour J. C. Colson P. Fournier P. E. Sallah K. Raoult D. (2015). A comprehensive repertoire of prokaryotic species identified in human beings. Lancet Infect. Dis. 15, 1211–1219. doi: 10.1016/S1473-3099(15)00293-5, PMID: 26311042

[ref73] Hung C. C. Chang C. C. Huang C. W. Nouchi R. Cheng C. H. (2022). Gut microbiota in patients with Alzheimer's disease spectrum: a systematic review and meta-analysis. Aging (Albany NY) 14, 477–496. doi: 10.18632/aging.203826, PMID: 35027502 PMC8791218

[ref74] Isacson R. Nielsen E. Dannaeus K. Bertilsson G. Patrone C. Zachrisson O.. (2011). The glucagon-like peptide 1 receptor agonist exendin-4 improves reference memory performance and decreases immobility in the forced swim test. Eur. J. Pharmacol. 650, 249–255. doi: 10.1016/j.ejphar.2010.10.008, PMID: 20951130

[ref75] Jia L. Du Y. Chu L. Zhang Z. Li F. Lyu D.. (2020). Prevalence, risk factors, and management of dementia and mild cognitive impairment in adults aged 60 years or older in China: a cross-sectional study. Lancet Public Health 5, e661–e671. doi: 10.1016/S2468-2667(20)30185-7, PMID: 33271079

[ref76] Johnson J. S. Spakowicz D. J. Hong B. Y. Petersen L. M. Demkowicz P. Chen L.. (2019). Evaluation of 16S rRNA gene sequencing for species and strain-level microbiome analysis. Nat. Commun. 10:5029. doi: 10.1038/s41467-019-13036-1 31695033 PMC6834636

[ref77] Justice N. J. (2018). The relationship between stress and Alzheimer's disease. Neurobiol Stress 8, 127–133. doi: 10.1016/j.ynstr.2018.04.002, PMID: 29888308 PMC5991350

[ref78] Karageorgis G. Warriner S. Nelson A. (2014). Efficient discovery of bioactive scaffolds by activity-directed synthesis. Nat. Chem. 6, 872–876. doi: 10.1038/nchem.2034, PMID: 25242481

[ref79] Kim C. S. Cha L. Sim M. Jung S. Chun W. Y. Baik H. W.. (2021). Probiotic supplementation improves cognitive function and mood with changes in gut microbiota in community-dwelling older adults: a randomized, double-blind, placebo-controlled, multicenter trial. J. Gerontol. A Biol. Sci. Med. Sci. 76, 32–40. doi: 10.1093/gerona/glaa090 32300799 PMC7861012

[ref80] Kim W. Egan J. M. (2008). The role of incretins in glucose homeostasis and diabetes treatment. Pharmacol. Rev. 60, 470–512. doi: 10.1124/pr.108.000604, PMID: 19074620 PMC2696340

[ref81] Kim D. S. Ko B. S. Ryuk J. A. Park S. (2020). *Tetragonia tetragonioides* protected against memory dysfunction by elevating hippocampal amyloid-beta deposition through potentiating insulin signaling and altering gut microbiome composition. Int. J. Mol. Sci. 21:2900. doi: 10.3390/ijms21249633, PMID: 32326255 PMC7216031

[ref82] Knopman D. S. Amieva H. Petersen R. C. Chetelat G. Holtzman D. M. Hyman B. T.. (2021). Alzheimer disease. Nat. Rev. Dis. Primers. 7:33. doi: 10.1038/s41572-021-00269-y 33986301 PMC8574196

[ref83] Kobayashi Y. Sugahara H. Shimada K. Mitsuyama E. Kuhara T. Yasuoka A.. (2017). Therapeutic potential of *Bifidobacterium breve* strain A1 for preventing cognitive impairment in Alzheimer's disease. Sci. Rep. 7:13510. doi: 10.1038/s41598-017-13368-2 29044140 PMC5647431

[ref84] Koh A. De Vadder F. Kovatcheva-Datchary P. Backhed F. (2016). From dietary Fiber to host physiology: short-chain fatty acids as key bacterial metabolites. Cells 165, 1332–1345. doi: 10.1016/j.cell.2016.05.041, PMID: 27259147

[ref85] Kowalski K. Mulak A. (2019). Brain-gut-microbiota Axis in Alzheimer's disease. J Neurogastroenterol Motil 25, 48–60. doi: 10.5056/jnm18087, PMID: 30646475 PMC6326209

[ref86] Kurita N. Yamashiro K. Kuroki T. Tanaka R. Urabe T. Ueno Y.. (2020). Metabolic endotoxemia promotes neuroinflammation after focal cerebral ischemia. J. Cereb. Blood Flow Metab. 40, 2505–2520. doi: 10.1177/0271678X19899577, PMID: 31910709 PMC7820690

[ref87] Larraufie P. Martin-Gallausiaux C. Lapaque N. Dore J. Gribble F. M. Reimann F.. (2018). SCFAs strongly stimulate PYY production in human enteroendocrine cells. Sci. Rep. 8:74. doi: 10.1038/s41598-017-18259-0 29311617 PMC5758799

[ref88] Lawson L. J. Perry V. H. Dri P. Gordon S. (1990). Heterogeneity in the distribution and morphology of microglia in the normal adult mouse brain. Neuroscience 39, 151–170. doi: 10.1016/0306-4522(90)90229-W, PMID: 2089275

[ref89] Lee J. Y. Hall J. A. Kroehling L. Wu L. Najar T. Nguyen H. H.. (2020). Serum amyloid a proteins induce pathogenic Th17 cells and promote inflammatory disease. Cells 180:e16. doi: 10.1016/j.cell.2019.11.026 PMC7039443 31866067

[ref90] Lee K. E. Kim J. K. Han S. K. Lee D. Y. Lee H. J. Yim S. V.. (2020). The extracellular vesicle of gut microbial *Paenalcaligenes hominis* is a risk factor for vagus nerve-mediated cognitive impairment. Microbiome 8:107. doi: 10.1186/s40168-020-00881-2 32669127 PMC7364628

[ref91] Levy M. Thaiss C. A. Zeevi D. Dohnalova L. Zilberman-Schapira G. Mahdi J. A.. (2015). Microbiota-modulated metabolites shape the intestinal microenvironment by regulating NLRP6 Inflammasome signaling. Cells 163, 1428–1443. doi: 10.1016/j.cell.2015.10.048, PMID: 26638072 PMC5665753

[ref92] Li Y. Hao Y. Zhu J. Owyang C. (2000). Serotonin released from intestinal enterochromaffin cells mediates luminal non-cholecystokinin-stimulated pancreatic secretion in rats. Gastroenterology 118, 1197–1207. doi: 10.1016/S0016-5085(00)70373-8, PMID: 10833495

[ref93] Li B. He Y. Ma J. Huang P. Du J. Cao L.. (2019). Mild cognitive impairment has similar alterations as Alzheimer's disease in gut microbiota. Alzheimers Dement. 15, 1357–1366. doi: 10.1016/j.jalz.2019.07.002 31434623

[ref94] Li H. Sun J. Wang F. Ding G. Chen W. Fang R.. (2016). Sodium butyrate exerts neuroprotective effects by restoring the blood-brain barrier in traumatic brain injury mice. Brain Res. 1642, 70–78. doi: 10.1016/j.brainres.2016.03.031, PMID: 27017959

[ref95] Li Z. Zeng Q. Hu S. Liu Z. Wang S. Jin Y.. (2023a). Chaihu Shugan san ameliorated cognitive deficits through regulating gut microbiota in senescence-accelerated mouse prone 8. Front. Pharmacol. 14:1181226. doi: 10.3389/fphar.2023.1181226 37256236 PMC10226648

[ref96] Li Z. Zhao T. Shi M. Wei Y. Huang X. Shen J.. (2023b). Polyphenols: natural food grade biomolecules for treating neurodegenerative diseases from a multi-target perspective. Front. Nutr. 10:1139558. doi: 10.3389/fnut.2023.1139558 36925964 PMC10011110

[ref97] Liu Y. Du T. Zhang W. Lu W. Peng Z. Huang S.. (2019). Modified Huang-Lian-Jie-Du decoction ameliorates Aβ Synaptotoxicity in a murine model of Alzheimer's disease. Oxidative Med. Cell. Longev. 2019:8340192. doi: 10.1155/2019/8340192, PMID: 31781354 PMC6875425

[ref98] Liu Y. J. Tang B. Wang F. C. Tang L. Lei Y. Y. Luo Y.. (2020). Parthenolide ameliorates colon inflammation through regulating Treg/Th17 balance in a gut microbiota-dependent manner. Theranostics 10, 5225–5241. doi: 10.7150/thno.43716, PMID: 32373209 PMC7196297

[ref99] Long J. M. Holtzman D. M. (2019). Alzheimer disease: an update on pathobiology and treatment strategies. Cells 179, 312–339. doi: 10.1016/j.cell.2019.09.001, PMID: 31564456 PMC6778042

[ref100] Loubinoux J. Bronowicki J. P. Pereira I. A. Mougenel J. L. Faou A. E. (2002). Sulfate-reducing bacteria in human feces and their association with inflammatory bowel diseases. FEMS Microbiol. Ecol. 40, 107–112. doi: 10.1111/j.1574-6941.2002.tb00942.x, PMID: 19709217

[ref101] Lu J. Guo P. Liu X. Zhang Y. Guo X. Gao X.. (2019). Herbal formula Fo Shou san attenuates Alzheimer's disease-related pathologies via the gut-liver-brain Axis in APP/PS1 mouse model of Alzheimer's disease. Evid. Based Complement. Alternat. Med. 2019:8302950. doi: 10.1155/2019/8302950, PMID: 31316576 PMC6601474

[ref102] Lupp C. Robertson M. L. Wickham M. E. Sekirov I. Champion O. L. Gaynor E. C.. (2007). Host-mediated inflammation disrupts the intestinal microbiota and promotes the overgrowth of Enterobacteriaceae. Cell Host Microbe 2, 119–129. doi: 10.1016/j.chom.2007.06.010, PMID: 18005726

[ref103] Lv M. Yang S. Cai L. Qin L. Q. Li B. Y. Wan Z. (2018). Effects of quercetin intervention on cognition function in APP/PS1 mice was affected by vitamin D status. Mol. Nutr. Food Res. 62:e1800621. doi: 10.1002/mnfr.201800621, PMID: 30328681

[ref104] Mahmoudiandehkordi S. Arnold M. Nho K. Ahmad S. Jia W. Xie G.. (2019). Altered bile acid profile associates with cognitive impairment in Alzheimer's disease-an emerging role for gut microbiome. Alzheimers Dement. 15, 76–92. doi: 10.1016/j.jalz.2018.07.217 30337151 PMC6487485

[ref105] Marks P. A. Richon V. M. Miller T. Kelly W. K. (2004). Histone deacetylase inhibitors. Adv. Cancer Res. 91, 137–168. 15327890 10.1016/S0065-230X(04)91004-4

[ref106] Matheson J. T. Holsinger R. M. D. (2023). The role of fecal microbiota transplantation in the treatment of neurodegenerative diseases: a review. Int. J. Mol. Sci. 24:1001. doi: 10.3390/ijms24021001, PMID: 36674517 PMC9864694

[ref107] Mcclean P. L. Parthsarathy V. Faivre E. Hölscher C. (2011). The diabetes drug liraglutide prevents degenerative processes in a mouse model of Alzheimer's disease. J. Neurosci. 31, 6587–6594. doi: 10.1523/JNEUROSCI.0529-11.2011, PMID: 21525299 PMC6622662

[ref108] Meng X. He M. Guo R. Duan R. Huo F. Lv C.. (2017). Investigation of the effect of the degree of processing of Radix Rehmanniae Preparata (Shu Dihuang) on Shu Dihuangtan carbonization preparation technology. Molecules 22:1193. doi: 10.3390/molecules22071193, PMID: 28718784 PMC6152270

[ref109] Meng F. Li N. Li D. Song B. Li L. (2019). The presence of elevated circulating trimethylamine N-oxide exaggerates postoperative cognitive dysfunction in aged rats. Behav. Brain Res. 368:111902. doi: 10.1016/j.bbr.2019.111902, PMID: 30980850

[ref110] Miller A. H. Raison C. L. (2016). The role of inflammation in depression: from evolutionary imperative to modern treatment target. Nat. Rev. Immunol. 16, 22–34. doi: 10.1038/nri.2015.5, PMID: 26711676 PMC5542678

[ref111] Milligan Armstrong A. Porter T. Quek H. White A. Haynes J. Jackaman C.. (2021). Chronic stress and Alzheimer's disease: the interplay between the hypothalamic-pituitary-adrenal axis, genetics and microglia. Biol. Rev. Camb. Philos. Soc. 96, 2209–2228. doi: 10.1111/brv.12750, PMID: 34159699

[ref112] Minter M. R. Zhang C. Leone V. Ringus D. L. Zhang X. Oyler-Castrillo P.. (2016). Antibiotic-induced perturbations in gut microbial diversity influences neuro-inflammation and amyloidosis in a murine model of Alzheimer's disease. Sci. Rep. 6:30028. doi: 10.1038/srep30028 27443609 PMC4956742

[ref113] Mitew S. Kirkcaldie M. T. Dickson T. C. Vickers J. C. (2013). Altered synapses and gliotransmission in Alzheimer's disease and AD model mice. Neurobiol. Aging 34, 2341–2351. doi: 10.1016/j.neurobiolaging.2013.04.010, PMID: 23643146

[ref114] Morgese M. G. Schiavone S. Trabace L. (2017). Emerging role of amyloid beta in stress response: implication for depression and diabetes. Eur. J. Pharmacol. 817, 22–29. doi: 10.1016/j.ejphar.2017.08.031, PMID: 28844871

[ref115] Mou Y. Du Y. Zhou L. Yue J. Hu X. Liu Y.. (2022). Gut microbiota interact with the brain through systemic chronic inflammation: implications on Neuroinflammation, neurodegeneration, and aging. Front. Immunol. 13:796288. doi: 10.3389/fimmu.2022.796288, PMID: 35464431 PMC9021448

[ref116] Mucke L. Masliah E. Yu G. Q. Mallory M. Rockenstein E. M. Tatsuno G.. (2000). High-level neuronal expression of abeta 1-42 in wild-type human amyloid protein precursor transgenic mice: synaptotoxicity without plaque formation. J. Neurosci. 20, 4050–4058. doi: 10.1523/JNEUROSCI.20-11-04050.2000, PMID: 10818140 PMC6772621

[ref117] Mucke L. Selkoe D. J. (2012). Neurotoxicity of amyloid beta-protein: synaptic and network dysfunction. Cold Spring Harb. Perspect. Med. 2:a006338. doi: 10.1101/cshperspect.a006338, PMID: 22762015 PMC3385944

[ref118] Murray M. M. Bernstein S. L. Nyugen V. Condron M. M. Teplow D. B. Bowers M. T. (2009). Amyloid beta protein: Abeta40 inhibits Abeta42 oligomerization. J. Am. Chem. Soc. 131, 6316–6317. doi: 10.1021/ja8092604, PMID: 19385598 PMC2697393

[ref119] Nagpal R. Neth B. J. Wang S. Craft S. Yadav H. (2019). Modified Mediterranean-ketogenic diet modulates gut microbiome and short-chain fatty acids in association with Alzheimer's disease markers in subjects with mild cognitive impairment. EBioMedicine 47, 529–542. doi: 10.1016/j.ebiom.2019.08.032, PMID: 31477562 PMC6796564

[ref120] Nowell J. Blunt E. Edison P. (2023). Incretin and insulin signaling as novel therapeutic targets for Alzheimer's and Parkinson's disease. Mol. Psychiatry 28, 217–229. doi: 10.1038/s41380-022-01792-4, PMID: 36258018 PMC9812772

[ref121] Nunes A. F. Amaral J. D. Lo A. C. Fonseca M. B. Viana R. J. Callaerts-Vegh Z. (2012). TUDCA, a bile acid, attenuates amyloid precursor protein processing and amyloid-beta deposition in APP/PS1 mice. Mol. Neurobiol. 45, 440–454. doi: 10.1007/s12035-012-8256-y, PMID: 22438081

[ref122] Orsini F. De Blasio D. Zangari R. Zanier E. R. De Simoni M. G. (2014). Versatility of the complement system in neuroinflammation, neurodegeneration and brain homeostasis. Front. Cell. Neurosci. 8:380. doi: 10.3389/fncel.2014.00380, PMID: 25426028 PMC4224073

[ref123] Palop J. J. Chin J. Roberson E. D. Wang J. Thwin M. T. Bien-Ly N.. (2007). Aberrant excitatory neuronal activity and compensatory remodeling of inhibitory hippocampal circuits in mouse models of Alzheimer's disease. Neuron 55, 697–711. doi: 10.1016/j.neuron.2007.07.025, PMID: 17785178 PMC8055171

[ref124] Park J. Kim M. Kang S. G. Jannasch A. H. Cooper B. Patterson J.. (2015). Short-chain fatty acids induce both effector and regulatory T cells by suppression of histone deacetylases and regulation of the mTOR-S6K pathway. Mucosal Immunol. 8, 80–93. doi: 10.1038/mi.2014.44, PMID: 24917457 PMC4263689

[ref125] Patrick S. (2015). “Chapter 51 - Bacteroides” in Molecular Medical Microbiology. eds. Tang Y.-W. Sussman M. Liu D. Poxton I. Schwartzman J.. Second ed (Boston: Academic Press), 917–944.

[ref126] Paula-Lima A. C. Brito-Moreira J. Ferreira S. T. (2013). Deregulation of excitatory neurotransmission underlying synapse failure in Alzheimer's disease. J. Neurochem. 126, 191–202. doi: 10.1111/jnc.12304, PMID: 23668663

[ref127] Pivovarova O. Hohn A. Grune T. Pfeiffer A. F. Rudovich N. (2016). Insulin-degrading enzyme: new therapeutic target for diabetes and Alzheimer's disease? Ann. Med. 48, 614–624. doi: 10.1080/07853890.2016.1197416, PMID: 27320287

[ref128] Porter D. W. Irwin N. Flatt P. R. Hölscher C. Gault V. A. (2011). Prolonged GIP receptor activation improves cognitive function, hippocampal synaptic plasticity and glucose homeostasis in high-fat fed mice. Eur. J. Pharmacol. 650, 688–693. doi: 10.1016/j.ejphar.2010.10.059, PMID: 21050845

[ref129] Psichas A. Sleeth M. L. Murphy K. G. Brooks L. Bewick G. A. Hanyaloglu A. C.. (2015). The short chain fatty acid propionate stimulates GLP-1 and PYY secretion via free fatty acid receptor 2 in rodents. Int. J. Obes. 39, 424–429. doi: 10.1038/ijo.2014.153, PMID: 25109781 PMC4356745

[ref130] Quinn M. Mcmillin M. Galindo C. Frampton G. Pae H. Y. Demorrow S. (2014). Bile acids permeabilize the blood brain barrier after bile duct ligation in rats via Rac1-dependent mechanisms. Dig. Liver Dis. 46, 527–534. doi: 10.1016/j.dld.2014.01.159, PMID: 24629820 PMC4065628

[ref131] Rajilic-Stojanovic M. Smidt H. De Vos W. M. (2007). Diversity of the human gastrointestinal tract microbiota revisited. Environ. Microbiol. 9, 2125–2136. doi: 10.1111/j.1462-2920.2007.01369.x, PMID: 17686012

[ref132] Ressler K. J. Mayberg H. S. (2007). Targeting abnormal neural circuits in mood and anxiety disorders: from the laboratory to the clinic. Nat. Neurosci. 10, 1116–1124. doi: 10.1038/nn1944, PMID: 17726478 PMC2444035

[ref133] Rhea E. M. Rask-Madsen C. Banks W. A. (2018). Insulin transport across the blood-brain barrier can occur independently of the insulin receptor. J. Physiol. 596, 4753–4765. doi: 10.1113/JP276149, PMID: 30044494 PMC6166047

[ref134] Rhee S. H. (2014). Lipopolysaccharide: basic biochemistry, intracellular signaling, and physiological impacts in the gut. Intest Res 12, 90–95. doi: 10.5217/ir.2014.12.2.90 25349574 PMC4204704

[ref135] Richards L. J. Chover-Gonzalez A. Harbuz M. S. Jessop D. S. (2006). Protective effects of endotoxin in a rat model of chronic inflammation are accompanied by suppressed secretion of pro-inflammatory cytokines and biphasic alteration in hypothalamo-pituitary-adrenal axis activity. J. Neuroendocrinol. 18, 875–882. doi: 10.1111/j.1365-2826.2006.01486.x, PMID: 17026537

[ref136] Roman G. C. Jackson R. E. Gadhia R. Roman A. N. Reis J. (2019). Mediterranean diet: the role of long-chain omega-3 fatty acids in fish; polyphenols in fruits, vegetables, cereals, coffee, tea, cacao and wine; probiotics and vitamins in prevention of stroke, age-related cognitive decline, and Alzheimer disease. Rev. Neurol. (Paris) 175, 724–741. doi: 10.1016/j.neurol.2019.08.005, PMID: 31521398

[ref137] Rothhammer V. Mascanfroni I. D. Bunse L. Takenaka M. C. Kenison J. E. Mayo L.. (2016). Type I interferons and microbial metabolites of tryptophan modulate astrocyte activity and central nervous system inflammation via the aryl hydrocarbon receptor. Nat. Med. 22, 586–597. doi: 10.1038/nm.4106, PMID: 27158906 PMC4899206

[ref138] Sagare A. P. Bell R. D. Zlokovic B. V. (2012). Neurovascular dysfunction and faulty amyloid beta-peptide clearance in Alzheimer disease. Cold Spring Harb. Perspect. Med. 2:11452. doi: 10.1101/cshperspect.a011452, PMID: 23028132 PMC3475405

[ref139] Saiyasit N. Chunchai T. Prus D. Suparan K. Pittayapong P. Apaijai N.. (2020). Gut dysbiosis develops before metabolic disturbance and cognitive decline in high-fat diet-induced obese condition. Nutrition 69:110576. doi: 10.1016/j.nut.2019.110576, PMID: 31580986

[ref140] Salyers A. A. (1984). Bacteroides of the human lower intestinal tract. Annu. Rev. Microbiol. 38, 293–313. doi: 10.1146/annurev.mi.38.100184.001453, PMID: 6388494

[ref141] Schroeder B. O. Backhed F. (2016). Signals from the gut microbiota to distant organs in physiology and disease. Nat. Med. 22, 1079–1089. doi: 10.1038/nm.4185, PMID: 27711063

[ref142] Selkoe D. J. (1991). The molecular pathology of Alzheimer's disease. Neuron 6, 487–498. doi: 10.1016/0896-6273(91)90052-2, PMID: 1673054

[ref143] Selkoe D. J. Hardy J. (2016). The amyloid hypothesis of Alzheimer's disease at 25 years. EMBO Mol. Med. 8, 595–608. doi: 10.15252/emmm.201606210, PMID: 27025652 PMC4888851

[ref144] Shaughness M. Acs D. Brabazon F. Hockenbury N. Byrnes K. R. (2020). Role of insulin in Neurotrauma and neurodegeneration: a review. Front. Neurosci. 14:547175. doi: 10.3389/fnins.2020.547175, PMID: 33100956 PMC7546823

[ref145] Shi J. Chen J. Xie X. Li Y. Ye W. Yao J.. (2023). Baicalein-corrected gut microbiota may underlie the amelioration of memory and cognitive deficits in APP/PS1 mice. Front. Pharmacol. 14:1132857. doi: 10.3389/fphar.2023.1132857 37063260 PMC10101436

[ref146] Shkoporov A. N. Khokhlova E. V. Kulagina E. V. Smeianov V. V. Kafarskaia L. I. Efimov B. A. (2008). Application of several molecular techniques to study numerically predominant Bifidobacterium spp. and Bacteroidales order strains in the feces of healthy children. Biosci. Biotechnol. Biochem. 72, 742–748. doi: 10.1271/bbb.70628 18323636

[ref147] Solch R. J. Aigbogun J. O. Voyiadjis A. G. Talkington G. M. Darensbourg R. M. O'connell S.. (2022). Mediterranean diet adherence, gut microbiota, and Alzheimer's or Parkinson's disease risk: a systematic review. J. Neurol. Sci. 434:120166. doi: 10.1016/j.jns.2022.120166 35144237

[ref148] Soldano A. Hassan B. A. (2014). Beyond pathology: APP, brain development and Alzheimer's disease. Curr. Opin. Neurobiol. 27, 61–67. doi: 10.1016/j.conb.2014.02.003, PMID: 24632309

[ref149] Sorboni S. G. Moghaddam H. S. Jafarzadeh-Esfehani R. Soleimanpour S. (2022). A comprehensive review on the role of the gut microbiome in human neurological disorders. Clin. Microbiol. Rev. 35:e0033820. doi: 10.1128/CMR.00338-20, PMID: 34985325 PMC8729913

[ref150] Stafford J. M. Raybuck J. D. Ryabinin A. E. Lattal K. M. (2012). Increasing histone acetylation in the hippocampus-infralimbic network enhances fear extinction. Biol. Psychiatry 72, 25–33. doi: 10.1016/j.biopsych.2011.12.012, PMID: 22290116 PMC3352991

[ref151] Strati F. Cavalieri D. Albanese D. De Felice C. Donati C. Hayek J.. (2017). New evidences on the altered gut microbiota in autism spectrum disorders. Microbiome 5:24. doi: 10.1186/s40168-017-0242-1 28222761 PMC5320696

[ref152] Su Y. Liu N. Sun R. Ma J. Li Z. Wang P.. (2023). Radix Rehmanniae Praeparata (Shu Dihuang) exerts neuroprotective effects on ICV-STZ-induced Alzheimer's disease mice through modulation of INSR/IRS-1/AKT/GSK-3beta signaling pathway and intestinal microbiota. Front. Pharmacol. 14:1115387. doi: 10.3389/fphar.2023.1115387 36843923 PMC9945319

[ref153] Subhramanyam C. S. Wang C. Hu Q. Dheen S. T. (2019). Microglia-mediated neuroinflammation in neurodegenerative diseases. Semin. Cell Dev. Biol. 94, 112–120. doi: 10.1016/j.semcdb.2019.05.004, PMID: 31077796

[ref154] Sun Z. Z. Li X. Y. Wang S. Shen L. Ji H. F. (2020). Bidirectional interactions between curcumin and gut microbiota in transgenic mice with Alzheimer's disease. Appl. Microbiol. Biotechnol. 104, 3507–3515. doi: 10.1007/s00253-020-10461-x 32095862

[ref155] Sun J. Xu J. Ling Y. Wang F. Gong T. Yang C.. (2019). Fecal microbiota transplantation alleviated Alzheimer's disease-like pathogenesis in APP/PS1 transgenic mice. Transl. Psychiatry 9:189. doi: 10.1038/s41398-019-0525-3 31383855 PMC6683152

[ref156] Sun J. Xu J. Yang B. Chen K. Kong Y. Fang N.. (2020). Effect of *Clostridium butyricum* against microglia-mediated Neuroinflammation in Alzheimer's disease via regulating gut microbiota and metabolites butyrate. Mol. Nutr. Food Res. 64:e1900636. doi: 10.1002/mnfr.201900636 31835282

[ref157] Szablewski L. (2018). Human gut microbiota in health and Alzheimer's disease. J. Alzheimers Dis. 62, 549–560. doi: 10.3233/JAD-170908, PMID: 29480188

[ref158] Szaruga M. Munteanu B. Lismont S. Veugelen S. Horre K. Mercken M.. (2017). Alzheimer's-causing mutations shift Abeta length by destabilizing gamma-secretase-Abetan interactions. Cells 170:e414. doi: 10.1016/j.cell.2021.03.058 28753424

[ref159] Tang W. H. Wang Z. Levison B. S. Koeth R. A. Britt E. B. Fu X.. (2013). Intestinal microbial metabolism of phosphatidylcholine and cardiovascular risk. N. Engl. J. Med. 368, 1575–1584. doi: 10.1056/NEJMoa1109400, PMID: 23614584 PMC3701945

[ref160] Thaler J. P. Yi C. X. Schur E. A. Guyenet S. J. Hwang B. H. Dietrich M. O.. (2012). Obesity is associated with hypothalamic injury in rodents and humans. J. Clin. Invest. 122, 153–162. doi: 10.1172/JCI59660, PMID: 22201683 PMC3248304

[ref161] Tolhurst G. Heffron H. Lam Y. S. Parker H. E. Habib A. M. Diakogiannaki E.. (2012). Short-chain fatty acids stimulate glucagon-like peptide-1 secretion via the G-protein-coupled receptor FFAR2. Diabetes 61, 364–371. doi: 10.2337/db11-1019, PMID: 22190648 PMC3266401

[ref162] Vakharia K. Hinson J. P. (2005). Lipopolysaccharide directly stimulates cortisol secretion by human adrenal cells by a cyclooxygenase-dependent mechanism. Endocrinology 146, 1398–1402. doi: 10.1210/en.2004-0882, PMID: 15564329

[ref163] Vargas-Caballero M. Warming H. Walker R. Holmes C. Cruickshank G. Patel B. (2022). Vagus nerve stimulation as a potential therapy in early Alzheimer's disease: a review. Front. Hum. Neurosci. 16:866434. doi: 10.3389/fnhum.2022.866434, PMID: 35572001 PMC9098960

[ref164] Verhaar B. J. H. Hendriksen H. M. A. De Leeuw F. A. Doorduijn A. S. Van Leeuwenstijn M. Teunissen C. E.. (2021). Gut microbiota composition is related to AD pathology. Front. Immunol. 12:794519. doi: 10.3389/fimmu.2021.794519, PMID: 35173707 PMC8843078

[ref165] Vogt N. M. Kerby R. L. Dill-Mcfarland K. A. Harding S. J. Merluzzi A. P. Johnson S. C.. (2017). Gut microbiome alterations in Alzheimer's disease. Sci. Rep. 7:13537. doi: 10.1038/s41598-017-13601-y 29051531 PMC5648830

[ref166] Volmar C.-H. Wahlestedt C. (2015). Histone deacetylases (HDACs) and brain function. Neuroepigenetics 1, 20–27. doi: 10.1016/j.nepig.2014.10.002, PMID: 37523880

[ref167] Wall R. Fitzgerald G. Hussey S. Ryan T. Murphy B. Ross P.. (2007). Genomic diversity of cultivable Lactobacillus populations residing in the neonatal and adult gastrointestinal tract. FEMS Microbiol. Ecol. 59, 127–137. doi: 10.1111/j.1574-6941.2006.00202.x 16978242

[ref168] Wang Q. Duan L. Li X. Wang Y. Guo W. Guan F.. (2022). Glucose metabolism, neural cell senescence and Alzheimer's disease. Int. J. Mol. Sci. 23:4351. doi: 10.3390/ijms23084351 35457168 PMC9030802

[ref169] Wang X. Heazlewood S. P. Krause D. O. Florin T. H. (2003). Molecular characterization of the microbial species that colonize human ileal and colonic mucosa by using 16S rDNA sequence analysis. J. Appl. Microbiol. 95, 508–520. doi: 10.1046/j.1365-2672.2003.02005.x 12911699

[ref170] Wang L. Hu Z. Yang W. Loo S. K. F. Ip S. P. Xian Y. F.. (2022). Anti-atopic dermatitis effect of a modified Huang-Lian-Jie-Du decoction and its active fraction on 2,4-dinitrobenzene and MC903-induced mouse models. Phytomedicine 104:154346. doi: 10.1016/j.phymed.2022.154346 35872445

[ref171] Wang S. Jiang W. Ouyang T. Shen X. Y. Wang F. Qu Y. H.. (2019). Jatrorrhizine balances the gut microbiota and reverses learning and memory deficits in APP/PS1 transgenic mice. Sci. Rep. 9:19575. doi: 10.1038/s41598-019-56149-9 31862965 PMC6925119

[ref172] Wang J. Lei X. Xie Z. Zhang X. Cheng X. Zhou W.. (2019). CA-30, an oligosaccharide fraction derived from Liuwei Dihuang decoction, ameliorates cognitive deterioration via the intestinal microbiome in the senescence-accelerated mouse prone 8 strain. Aging (Albany NY) 11, 3463–3486. doi: 10.18632/aging.101990 31160541 PMC6594795

[ref173] Wang L. Lu J. Zeng Y. Guo Y. Wu C. Zhao H.. (2020). Improving Alzheimer's disease by altering gut microbiota in tree shrews with ginsenoside Rg1. FEMS Microbiol. Lett. 367:fnaa011. doi: 10.1093/femsle/fnaa011, PMID: 31950993

[ref174] Wang X. Sun G. Feng T. Zhang J. Huang X. Wang T.. (2019). Sodium oligomannate therapeutically remodels gut microbiota and suppresses gut bacterial amino acids-shaped neuroinflammation to inhibit Alzheimer's disease progression. Cell Res. 29, 787–803. doi: 10.1038/s41422-019-0216-x 31488882 PMC6796854

[ref175] Wang W. Y. Tan M. S. Yu J. T. Tan L. (2015). Role of pro-inflammatory cytokines released from microglia in Alzheimer's disease. Ann Transl Med 3:136. doi: 10.3978/j.issn.2305-5839.2015.03.49, PMID: 26207229 PMC4486922

[ref176] Wang Z. Tang W. H. Buffa J. A. Fu X. Britt E. B. Koeth R. A.. (2014). Prognostic value of choline and betaine depends on intestinal microbiota-generated metabolite trimethylamine-N-oxide. Eur. Heart J. 35, 904–910. doi: 10.1093/eurheartj/ehu002, PMID: 24497336 PMC3977137

[ref177] Wang Q. Yao H. Liu W. Ya B. Cheng H. Xing Z.. (2021). Microglia polarization in Alzheimer's disease: mechanisms and a potential therapeutic target. Front. Aging Neurosci. 13:772717. doi: 10.3389/fnagi.2021.772717 34819850 PMC8606412

[ref178] Wang J. Ye F. Cheng X. Zhang X. Liu F. Liu G.. (2016). The effects of LW-AFC on intestinal microbiome in senescence-accelerated mouse prone 8 strain, a mouse model of Alzheimer's disease. J. Alzheimers Dis. 53, 907–919. doi: 10.3233/JAD-160138, PMID: 27340848

[ref179] Wang H. Yu M. Ochani M. Amella C. A. Tanovic M. Susarla S.. (2003). Nicotinic acetylcholine receptor alpha7 subunit is an essential regulator of inflammation. Nature 421, 384–388. doi: 10.1038/nature01339 12508119

[ref180] Wang C. Zhao J. Zhang H. Lee Y. K. Zhai Q. Chen W. (2021). Roles of intestinal bacteroides in human health and diseases. Crit. Rev. Food Sci. Nutr. 61, 3518–3536. 32757948 10.1080/10408398.2020.1802695

[ref181] Wastyk H. C. Fragiadakis G. K. Perelman D. Dahan D. Merrill B. D. Yu F. B.. (2021). Gut-microbiota-targeted diets modulate human immune status. Cells 184:e4114. doi: 10.1016/j.cell.2021.06.019 PMC9020749 34256014

[ref182] Whitman W. B. Coleman D. C. Wiebe W. J. (1998). Prokaryotes: the unseen majority. Proc. Natl. Acad. Sci. U. S. A. 95, 6578–6583. doi: 10.1073/pnas.95.12.6578, PMID: 9618454 PMC33863

[ref183] Winston J. A. Theriot C. M. (2020). Diversification of host bile acids by members of the gut microbiota. Gut Microbes 11, 158–171. doi: 10.1080/19490976.2019.1674124, PMID: 31595814 PMC7053883

[ref184] Xie Z. Lu H. Yang S. Zeng Y. Li W. Wang L.. (2020). Salidroside attenuates cognitive dysfunction in senescence-accelerated mouse prone 8 (SAMP8) mice and modulates inflammation of the gut-brain Axis. Front. Pharmacol. 11:568423. doi: 10.3389/fphar.2020.568423, PMID: 33362539 PMC7759146

[ref185] Xin Y. Diling C. Tianlu C. Jun Z. Xiaocui T. Yinrui G.. (2019). Oligosaccharides from Morinda officinalis slow the Progress of aging mice by regulating the key microbiota-metabolite pairs. Evid. Based Complement. Alternat. Med. 2019:9306834. doi: 10.1155/2019/9306834, PMID: 31929824 PMC6942866

[ref186] Xu N. Huang F. Jian C. Qin L. Lu F. Wang Y.. (2019). Neuroprotective effect of salidroside against central nervous system inflammation-induced cognitive deficits: a pivotal role of sirtuin 1-dependent Nrf-2/HO-1/NF-kappaB pathway. Phytother. Res. 33, 1438–1447. doi: 10.1002/ptr.6335, PMID: 30848530

[ref187] Xu Y. Jiang C. Wu J. Liu P. Deng X. Zhang Y.. (2022). Ketogenic diet ameliorates cognitive impairment and neuroinflammation in a mouse model of Alzheimer's disease. CNS Neurosci. Ther. 28, 580–592. doi: 10.1111/cns.13779, PMID: 34889516 PMC8928920

[ref188] Yang S. Zhou H. Wang G. Zhong X. H. Shen Q. L. Zhang X. J.. (2020). Quercetin is protective against short-term dietary advanced glycation end products intake induced cognitive dysfunction in aged ICR mice. J. Food Biochem. 44:e13164. doi: 10.1111/jfbc.13164, PMID: 32065675

[ref189] Yankner B. Duffy L. Kirschner D. (1990). Neurotrophic and neurotoxic effects of amyloid β protein: reversal by tachykinin neuropeptides. Science (New York, N.Y.) 250, 279–282. 2218531 10.1126/science.2218531

[ref190] Yesiltepe M. Cimen B. Sara Y. (2022). Effects of chronic vagal nerve stimulation in the treatment of beta-amyloid-induced neuropsychiatric symptoms. Eur. J. Pharmacol. 931:175179. doi: 10.1016/j.ejphar.2022.175179, PMID: 35973478

[ref191] Yu L. C. Chen S. C. Chang W. C. Huang Y. C. Lin K. M. Lai P. H.. (2007). Stability of angiogenic agents, ginsenoside Rg1 and re, isolated from *Panax ginseng*: in vitro and in vivo studies. Int. J. Pharm. 328, 168–176. doi: 10.1016/j.ijpharm.2006.08.009 16962729

[ref192] Yue S. He T. Li B. Qu Y. Peng H. Chen J.. (2019). Effectiveness of Yi-Zhi-an-Shen granules on cognition and sleep quality in older adults with amnestic mild cognitive impairment: protocol for a randomized, double-blind, placebo-controlled trial. Trials 20:518. doi: 10.1186/s13063-019-3607-x 31429790 PMC6701140

[ref193] Zenaro E. Piacentino G. Constantin G. (2017). The blood-brain barrier in Alzheimer's disease. Neurobiol. Dis. 107, 41–56. doi: 10.1016/j.nbd.2016.07.007, PMID: 27425887 PMC5600438

[ref194] Zhang J. Ke K. F. Liu Z. Qiu Y. H. Peng Y. P. (2013). Th17 cell-mediated neuroinflammation is involved in neurodegeneration of abeta1-42-induced Alzheimer's disease model rats. PLoS One 8:e75786. doi: 10.1371/journal.pone.0085170, PMID: 24124514 PMC3790825

[ref195] Zhang B. Zhang Y. Wu W. Xu T. Yin Y. Zhang J.. (2017). Chronic glucocorticoid exposure activates BK-NLRP1 signal involving in hippocampal neuron damage. J. Neuroinflammation 14:139. doi: 10.1186/s12974-017-0911-9 28732502 PMC5521122

[ref196] Zhang X. Zhong H. Li Y. Shi Z. Ren H. Zhang Z.. (2021). Sex- and age-related trajectories of the adult human gut microbiota shared across populations of different ethnicities. Nature Aging 1, 87–100. doi: 10.1038/s43587-020-00014-2, PMID: 37118004

[ref197] Zhao Y. Cong L. Jaber V. Lukiw W. J. (2017a). Microbiome-derived lipopolysaccharide enriched in the perinuclear region of Alzheimer's disease brain. Front. Immunol. 8:1064. doi: 10.3389/fimmu.2017.01064 28928740 PMC5591429

[ref198] Zhao Y. Cong L. Lukiw W. J. (2017b). Lipopolysaccharide (LPS) accumulates in neocortical neurons of Alzheimer's disease (AD) brain and impairs transcription in human neuronal-glial primary co-cultures. Front. Aging Neurosci. 9:407. doi: 10.3389/fnagi.2017.00407 29311897 PMC5732913

[ref199] Zhao Y. Jaber V. Lukiw W. J. (2017c). Secretory products of the human GI tract microbiome and their potential impact on Alzheimer's disease (AD): detection of lipopolysaccharide (LPS) in AD Hippocampus. Front. Cell. Infect. Microbiol. 7:318. doi: 10.3389/fcimb.2017.00318 28744452 PMC5504724

[ref200] Zhao B. S. Liu Y. Gao X. Y. Zhai H. Q. Guo J. Y. Wang X. Y. (2014). Effects of ginsenoside Rg1 on the expression of toll-like receptor 3, 4 and their signalling transduction factors in the NG108-15 murine neuroglial cell line. Molecules 19, 16925–16936. doi: 10.3390/molecules191016925, PMID: 25340298 PMC6271333

[ref201] Zhao Y. Lukiw W. J. (2015). Microbiome-generated amyloid and potential impact on amyloidogenesis in Alzheimer's disease (AD). J. Nat. Sci. 1:e138. PMID: 26097896 PMC4469284

[ref202] Zhou H. Tai J. Xu H. Lu X. Meng D. (2019). Xanthoceraside could ameliorate Alzheimer's disease symptoms of rats by affecting the gut microbiota composition and modulating the endogenous metabolite levels. Front. Pharmacol. 10:1035. doi: 10.3389/fphar.2019.01035 31572201 PMC6753234

[ref203] Zott B. Simon M. M. Hong W. Unger F. Chen-Engerer H. J. Frosch M. P.. (2019). A vicious cycle of beta amyloid-dependent neuronal hyperactivation. Science 365, 559–565. doi: 10.1126/science.aay0198, PMID: 31395777 PMC6690382

